# A novel pathogenic mutation of MeCP2 impairs chromatin association independent of protein levels

**DOI:** 10.1101/gad.350733.123

**Published:** 2023-10-01

**Authors:** Jian Zhou, Claudia Cattoglio, Yingyao Shao, Harini P. Tirumala, Carlo Vetralla, Sameer S. Bajikar, Yan Li, Hu Chen, Qi Wang, Zhenyu Wu, Bing Tang, Mahla Zahabiyon, Aleksandar Bajic, Xiangling Meng, Jack J. Ferrie, Anel LaGrone, Ping Zhang, Jean J. Kim, Jianrong Tang, Zhandong Liu, Xavier Darzacq, Nathaniel Heintz, Robert Tjian, Huda Y. Zoghbi

**Affiliations:** 1 Department of Molecular and Human Genetics, Baylor College of Medicine, Houston, Texas 77030, USA;; 2 Jan and Dan Duncan Neurological Research Institute at Texas Children's Hospital, Houston, Texas 77030, USA;; 3 Department of Molecular and Cell Biology, Li Ka Shing Center for Biomedical and Health Sciences, California Institute for Regenerative Medicine (CIRM) Center of Excellence, University of California, Berkeley, Berkeley, California 94720, USA;; 4 Howard Hughes Medical Institute, Berkeley, California 94720, USA;; 5 Program in Developmental Biology, Baylor College of Medicine, Houston, Texas 77030, USA;; 6 School of Medicine and Surgery, University of Milan-Bicocca, Milano 20126, Italy;; 7 Department of Pediatrics, Baylor College of Medicine, Houston, Texas 77030, USA;; 8 Department of Neuroscience, Baylor College of Medicine, Houston, Texas 77030, USA;; 9 Advanced Technology Cores, Baylor College of Medicine, Houston, Texas 77030, USA;; 10 Department of Molecular and Cellular Biology, Stem Cells and Regenerative Medicine Center, Baylor College of Medicine, Houston, Texas 77030, USA;; 11 Laboratory of Molecular Biology, Howard Hughes Medical Institute, The Rockefeller University, New York, New York 10065, USA;; 12 Howard Hughes Medical Institute, Baylor College of Medicine, Houston, Texas 77030, USA

**Keywords:** chromatin dynamics, MeCP2, neurological disorders, Rett syndrome, single-molecular imaging

## Abstract

Loss-of-function mutations in *MECP2* cause Rett syndrome (RTT), a severe neurological disorder that mainly affects girls. Mutations in *MECP2* do occur in males occasionally and typically cause severe encephalopathy and premature lethality. Recently, we identified a missense mutation (c.353G>A, p.Gly118Glu [G118E]), which has never been seen before in *MECP2*, in a young boy who suffered from progressive motor dysfunction and developmental delay. To determine whether this variant caused the clinical symptoms and study its functional consequences, we established two disease models, including human neurons from patient-derived iPSCs and a knock-in mouse line. G118E mutation partially reduces MeCP2 abundance and its DNA binding, and G118E mice manifest RTT-like symptoms seen in the patient, affirming the pathogenicity of this mutation. Using live-cell and single-molecule imaging, we found that G118E mutation alters MeCP2's chromatin interaction properties in live neurons independently of its effect on protein levels. Here we report the generation and characterization of RTT models of a male hypomorphic variant and reveal new insight into the mechanism by which this pathological mutation affects MeCP2's chromatin dynamics. Our ability to quantify protein dynamics in disease models lays the foundation for harnessing high-resolution single-molecule imaging as the next frontier for developing innovative therapies for RTT and other diseases.

## Abstract (precis)

In this study, Zhou et al. characterize the molecular, behavioral, and physiological consequences of a novel loss-of-function G118E mutation in the methyl-CpG binding protein MECP2, identified in a patient with Rett syndrome. G118E diminished MECP2 protein levels and independently accelerated its nuclear mobility, altogether limiting MECP2's functional interaction with chromatin in neurons.

Rett syndrome (RTT) is a postnatal neurological disorder caused by loss-of-function mutations in the X-linked gene *methyl-CpG binding protein 2* (*MECP2*), with an approximate prevalence of one in 10,000 births (Amir et al. 1999; Laurvick et al. 2006). RTT is characterized by an initial 6–18 mo of normal development followed by progressive neurological dysfunction and developmental regression, including stereotypic hand-wringing, gait abnormalities, loss of speech, and deceleration of head growth (Chahrour and Zoghbi 2007). Severe loss-of-function mutations in *MECP2* cause RTT primarily in females, who are mosaic for cells that express either the wild-type or mutant version of MeCP2 due to random X-chromosome inactivation. Meanwhile, males with these same severe mutations rarely survive infancy because they lack a functional protein in all their cells (Neul et al. 2019). In rare cases, a small number of mutations in *MECP2*, such as A140V, cause mild loss of function and can be seen in males with intellectual disability and neuropsychiatric features (Winnepenninckx et al. 2002).

Many severe RTT-causing missense mutations in *MECP2*, such as R111G and R106W, cluster in the methyl-CpG binding domain (MBD) (Nan et al. 1993) and abolish MeCP2 binding to methylated cytosines (Goffin et al. 2012; Heckman et al. 2014; Brown et al. 2016; Johnson et al. 2017). Mouse models carrying these severe mutations have highlighted the importance of MeCP2 levels and its DNA binding for normal brain function (Guy et al. 2001; Goffin et al. 2012; Heckman et al. 2014; Brown et al. 2016; Johnson et al. 2017). Importantly, the majority (∼75%) of RTT cases are not caused by severe mutations that totally abolish MeCP2 function but rather by mutations that either reduce MeCP2 levels or DNA binding, leading to retention of some partial function of the protein. Thus, in principle, increasing the abundance or DNA binding ability of partially functional MeCP2 proteins should reduce the severity of RTT (Lamonica et al. 2017) in most cases, except those caused by mutations that lead to total loss of function. To this end, we reasoned that developing cellular and mouse models of a new *MECP2* mutation that does not totally inactivate the protein will help provide new high-resolution platforms to reliably quantify and compare phenotypic readouts that go beyond behavioral assessments to include DNA binding dynamics.

While DNA binding is essential for MeCP2 function, quantitative measurement of this property in live neurons under physiological conditions remains a challenge. Recently, live-cell imaging such as single-molecule tracking (SMT) of chromatin proteins has revealed important new insight into their dynamic behavior in the nucleus (Hansen et al. 2017; Chong et al. 2018; Liu and Tjian 2018). Indeed, previous SMT analysis showed that the R106W mutation, which completely abolishes MeCP2's DNA binding, dramatically altered the fraction of MeCP2 molecules bound to the chromatin and the diffusion of unbound molecules (Piccolo et al. 2019). However, no obvious abnormalities were detected in the milder R133C mutation that retains significant DNA binding capacity (Piccolo et al. 2019). It is thus important to evaluate whether a mutation that partially compromises DNA binding affects MeCP2's dynamic behavior in live neurons and whether SMT can reliably discriminate such pathological MeCP2 from wild-type MeCP2 under physiological conditions.

Here, we report a newly identified missense mutation in the MBD of *MECP2* found in a young boy with developmental delay, language and motor difficulties, and seizures. We generated disease models to establish the pathogenicity of this novel male RTT variant and performed single-molecule tracking, which revealed new insight into how this mutation affects MeCP2 chromatin binding dynamics.

## Results

### Identification of a male patient with a novel missense mutation in *MECP2* that affects protein level

**Figure 1.**
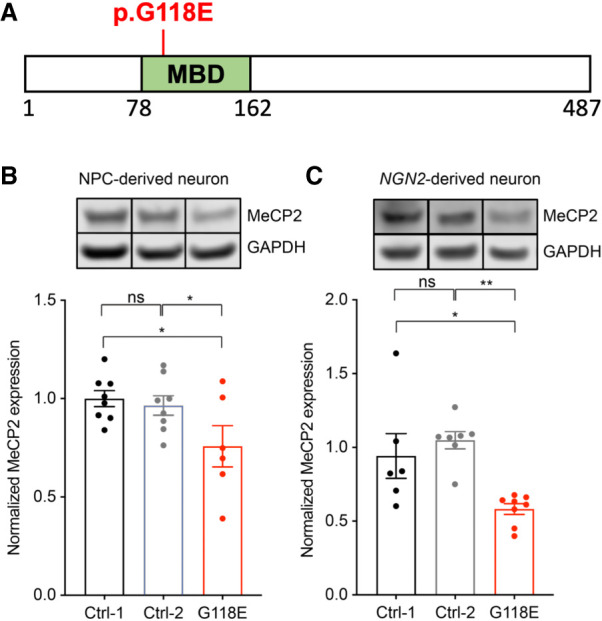
MeCP2 protein level is reduced in the patient fibroblast-derived neurons. (*A*) Diagram of MeCP2 protein with the G118E mutation in the methyl-CpG binding domain (MBD). (*B*) Western blot and quantification of MeCP2 protein levels in neurons derived from neural progenitor cells (*n* = 6–8). (*C*) Western blot and quantification of MeCP2 protein levels in neurons derived directly by NGN2 overexpression (*n* = 6–8). All data were analyzed by one-way ANOVA followed by Fisher's LSD post-hoc test. Data are presented as mean ± SEM. (*) *P* < 0.05, (**) *P* < 0.01, (ns.) not significant.

The patient is a young boy with a history of global developmental delay, generalized hypotonia, difficulty with motor planning, and metatarsus adductus. As he got older, he did develop some seizures and dystonia. Whole-exome sequencing from a buccal swab detected a de novo variant (c.353G>A, p.Gly118Glu [G118E]) in *MECP2*. We confirmed this mutation in his fibroblasts and did not observe mosaicism for the mutation. The G118E missense variant is predicted to change a highly conserved glycine to a glutamic acid in the MBD of the MeCP2 protein (Fig. 1A). This mutation is absent from the gnomAD, NHLBI Exome Sequencing Project, and 1000 Genomes databases, indicating that it is not a common benign variant in the populations represented therein. This novel *MECP2* mutation had not been identified in any RTT patient to date; as such, the functional consequences of this mutation were unknown.

To determine the effects of the G118E mutation on MeCP2 in patient cells, we generated iPSCs from the patient's fibroblasts. Additionally, we used CRISPR–Cas9 to edit the mutant nucleotide back to wild type (A > G), generating two isogenic control iPSC lines (Supplemental Fig. S1A). In these iPSC lines, *MECP2* RNA level was similar between the G118E sample and isogenic control (Supplemental Fig. S1B). We differentiated the iPSCs into neurons using two independent protocols to generate reliable results. In the first approach, we first differentiated the iPSCs into neuronal progenitor cells (Supplemental Fig. S2) and then into neurons. For the second approach, we directly differentiated the stem cells into neurons by Neurogenin-2 (*NGN2*) overexpression (Zhang et al. 2013). In both cases, the protein levels of MeCP2 were significantly reduced in G118E neurons (Fig. 1B,C; Supplemental Fig. S3A,B), confirming that the G118E mutation negatively affects MeCP2 protein levels in patient iPSC-derived neurons.

### Mecp2^G118E/y^ mice have reduced MeCP2 levels and partially impaired DNA binding

**Figure 2.**
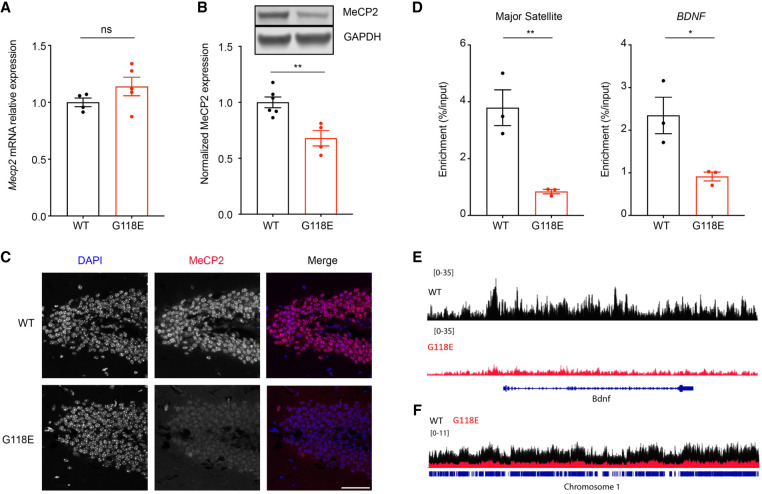
Reduced MeCP2 level and binding in *Mecp2*^*G118E/y*^ mice. (*A*) Quantitative PCR on *Mecp2* mRNA expression in the cortices of 8-wk-old WT and *Mecp2*^*G118E/y*^ mice (*n* = 4–5). (*B*) Western blot of MeCP2 protein level in 8-wk-old WT and *Mecp2*^*G118E/y*^ mice in the cortex (*n* = 4–6). (*C*) Immunofluorescence staining showed MeCP2 binding to heterochromatin foci in 8-wk-old WT and *Mecp2*^*G118E/y*^ mice in the hippocampus. Scale bar, 50 μm. (*D*) ChIP-qPCR of MeCP2 binding targets in 8-wk-old WT and *Mecp2*^*G118E/y*^ mice in the frontal cortex (*n* = 3). (*E*,*F*) CUT&RUN profiling of MeCP2 binding in WT and *Mecp2*^*G118E/y*^ mouse cortices at the *Bdnf* locus (*E*) and the entire chromosome 1 (*F*). Tracks from *Mecp2*^*G118E/y*^ tissues are colored red, and the scale of signal intensity is shown at the *top left* of each track. The displayed track is pooled from biological replicates (*n* = 3 biological replicates per genotype). All data were analyzed by unpaired *t*-test. Data are presented as mean ± SEM. (*) *P* < 0.05, (**) *P* < 0.01, (ns.) not significant.

To determine how the G118E mutation impacts MeCP2 level and function in vivo, we generated a knock-in mouse model of the G118E mutation using CRISPR–Cas9. Consistent with the G118E human iNeurons, *Mecp2*^*G118E/y*^ mice express *Mecp2* RNA at levels comparable with wild-type mice in the cortex (Fig. 2A) but have an ∼40% reduction in MeCP2 protein (Fig. 2B; Supplemental Fig. S3C). MeCP2 is a nuclear protein that binds to methylated cytosines (Meehan et al. 1992; Chen et al. 2015). Immunostaining confirmed a reduction in the level of MeCP2 protein localized in the heavily methylated heterochromatic foci in *Mecp2*^*G118E/y*^ mouse brains (Fig. 2C; Nan et al. 1996; Ito-Ishida et al. 2020). To assess MeCP2's chromatin binding, we performed ChIP-qPCR in *Mecp2*^*G118E/y*^ mouse frontal cortices and found that the binding of MeCP2 to its target genes is decreased (Fig. 2D). We next performed cleavage under targets and release using nuclease (CUT&RUN) to profile the genome-wide binding pattern of WT and G118E MeCP2 in mouse cortices. Consistent with the ChIP-qPCR result, the G118E mutation reduced MeCP2 binding to its target gene, *Bdnf* (Fig. 2E). In addition, we observed a genome-wide reduction of MeCP2's chromatin binding in *Mecp2*^*G118E/y*^ mice (Fig. 2F). These results indicate that the G118E mutation compromises MeCP2's chromatin binding globally, though we cannot decouple the decrease in binding from the overall reduced MeCP2 protein level.

### *Mecp2*^*G118E/y*^ mice show RTT-like behavioral phenotypes

RTT mouse models carrying strong loss-of-function alleles reproduce the symptoms seen in the RTT patients by displaying a wide array of behavioral deficits, including motor coordination problems, reduced anxiety, and impaired cued and context-dependent learning and memory (Guy et al. 2001; Chao et al. 2010; Samaco et al. 2013; Lavery et al. 2020). To determine whether the molecular changes caused by the G118E mutation results in a RTT-like phenotype in mice, we characterized the *Mecp2*^*G118E/y*^ mice using a series of behavioral assays.

**Figure 3.**
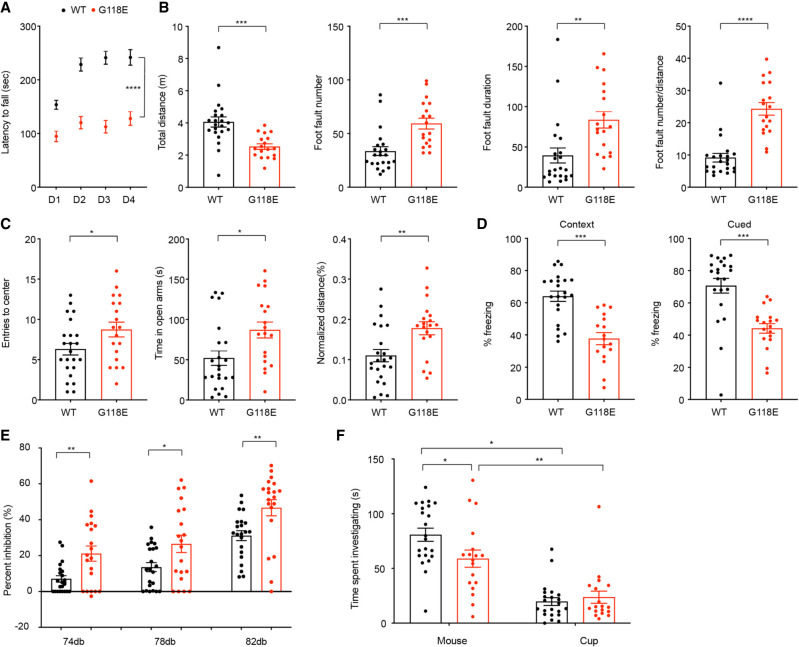
*Mecp2*^*G118E/y*^ mice show RTT-like behavioral phenotypes. (*A*) Rotarod assay tested the motor coordination and learning in 9-wk-old WT and *Mecp2*^*G118E/y*^ mice. (*B*) Parallel footslip assay showed the motor coordination in 12-wk-old WT and *Mecp2*^*G118E/y*^ mice. (*C*) Elevated plus maze measured the anxiety-like phenotypes in 10-wk-old WT and *Mecp2*^*G118E/y*^ mice. (*D*) Fear conditioning assay tested the contextual hippocampal and cued amygdala-dependent learning and memory in 12-wk-old WT and *Mecp2*^*G118E/y*^ mice. (*E*) Acoustic startle measured the sensorimotor gating in 10-wk-old WT and *Mecp2*^*G118E/y*^ mice. (*F*) Three-chamber assay showed the social interaction with either a cup or a partner mouse in 11-wk-old WT and *Mecp2*^*G118E/y*^ mice. *n* = 18–23 for all behavioral tests. All data were analyzed by unpaired *t*-test, except for the rotarod test, which was analyzed by two-way ANOVA with repeated measures. Data are presented as mean ± SEM. (*) *P* < 0.05, (**) *P* < 0.01, (***) *P* < 0.001, (****) *P* < 0.0001.

Motor impairment is the most prominent phenotype in RTT patients as well as the mouse models. Therefore, we tested motor coordination in *Mecp2*^*G118E/y*^ mice using rotarod and parallel footslip assays. *Mecp2*^*G118E/y*^ mice showed reduced latency on the accelerating rod compared with wild-type littermates in the rotarod test, and their performance did not improve over the 4 d of testing (Fig. 3A). In the parallel footslip assay, *Mecp2*^*G118E/y*^ mice displayed a higher number of footslips from the grid compared with wild-type animals (Fig. 3B), indicating impaired motor coordination and learning. To measure anxiety-like behavior, we performed an elevated plus maze assay and found that *Mecp2*^*G118E/y*^ mice display reduced anxiety compared with their wild-type littermates, as measured by increased entries to center, time spent in the open arms, and normalized distance in open arms (Fig. 3C). To test learning and memory in mice, we performed a fear conditioning assay. *Mecp2*^*G118E/y*^ mice showed deficits in both context and cued learning, as measured by reduced freezing (Fig. 3D). Additionally, *Mecp2*^*G118E/y*^ mice startled less and had enhanced prepulse inhibition, which is indicative of abnormalities in sensory function (Fig. 3E). Last, *Mecp2*^*G118E/y*^ mice displayed social deficits, as measured by decreased time investigating a partner mouse in the three-chamber assay (Fig. 3F). Taken together, these data suggest that *Mecp2*^*G118E/y*^ mice have RTT-like behavioral deficits that have been consistently observed in other *Mecp2* mutation-carrying mouse models.

### *Mecp2*^*G118E/y*^ mice display a range of RTT-like physiological abnormalities

**Figure 4.**
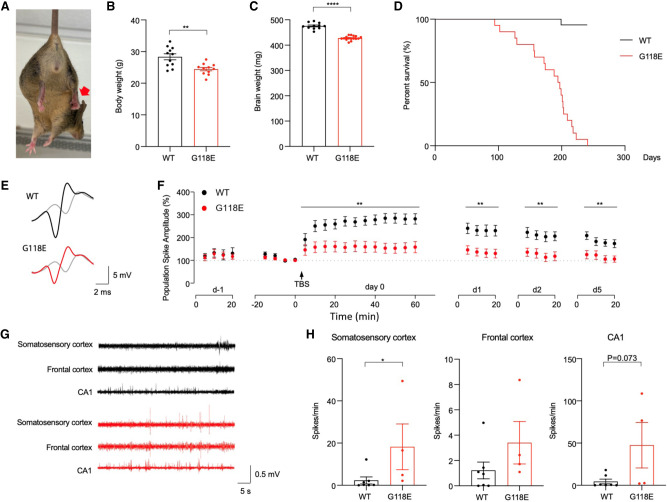
Physiological abnormalities and survival in *Mecp2*^*G118E/y*^ mice. (*A*) Hindlimb clasping was observed in 6-wk-old *Mecp2*^*G118E/y*^ mice. (*B*) *Mecp2*^*G118E/y*^ mice have lower body weight at 9 wk of age than the WT littermates (*n* = 11 per group). (**) *P* < 0.01, unpaired *t*-test. (*C*) *Mecp2*^*G118E/y*^ mice showed reduced brain weight (*n* = 11). (****) *P* < 0.0001, unpaired t-test. (*D*) Kaplan–Meier survival curve showing significantly reduced median life span of *Mecp2*^*G118E/y*^ mice compared with WT littermates (*n* = 10 per group). (*E*) Superimposed traces of the perforant path recorded in the dentate gyrus 5 min before (gray) and 55 min after LTP induction in WT (black) versus *Mecp2*^*G118E/y*^ (red) mice. (*F*) Summary of in vivo LTP in WT (*n* = 12) and *Mecp2*^*G118E/y*^ (*n* = 12) mice. LTP was induced by θ burst stimulation (TBS; arrow). Both groups showed LTP of population spike amplitudes (one-way repeated measures ANOVA on ranks on day 0; WT: *P* ≤ 0.001; mutant: *P* ≤ 0.001). Two-way repeated measures ANOVA revealed significant main effects of population spike amplitudes between the two groups on day 0 (genotype: *F*_1,22_ = 12.39, *P* = 0.002; time: *F*_11,242_ = 9.51, *P* < 0.001; genotype × time interaction: *F*_11,242_ = 6.72, *P* < 0.001), day 1 (genotype: *F*_1,22_ = 11.22, *P* = 0.003; time: *F*_3,66_ = 5.29, *P* = 0.002), day 2 (genotype: *F*_1,22_ = 10.34, *P* = 0.004; time: *F*_3,66_ = 3.66, *P* = 0.017), and day 5 (genotype: *F*_1,22_ = 10.27, *P* = 0.004; time: *F*_3,66_ = 7.01, *P* < 0.001) after induction. (**) *P* < 0.01. Data are presented as mean ± SEM. (*G*) Cortical EEG was recorded in the somatosensory cortex and the frontal cortex, while local field potentials were recorded in the CA1 region of the hippocampus. (*H*) The *Mecp2*^*G118E/y*^ mice (*n* = 4) showed more hyperexcitability discharges in the somatosensory cortex than the WT littermates (*n* = 7). No behavioral seizure or electrographic seizure was observed. Man–Whitney test, (*) *P* < 0.05. Data are presented as mean ± SEM.

To further understand the consequences of the G118E mutation, we characterized the physiology of *Mecp2*^*G118E/y*^ mice. By 8 wk of age, they developed hindlimb spasticity (Fig. 4A) and did not gain as much weight as wild-type mice starting from 9 wk (Fig. 4B). These mice also showed microcephaly (Fig. 4C), which is a common feature among RTT patients (Tarquinio et al. 2012), with no gross changes in brain anatomy and structure (Supplemental Fig. S4). They also had a reduced life span, with a median survival of ∼6 mo (Fig. 4D), which is quite longer than the *Mecp2*^*null*^, *Mecp2*^*T158M*^, and *Mecp2*^*R111G*^ mice but similar to the *Mecp2*^*R306C*^ mice and shorter than the *Mecp2*^*R133C*^ mice (Guy et al. 2007; Heckman et al. 2014; Brown et al. 2016). Furthermore, since we observed hippocampus-dependent learning and memory deficits in these mice, we examined in vivo long-term potentiation (LTP) to measure hippocampal synaptic plasticity, which serves as a neural substrate of learning and memory (Moser et al. 1998; Malenka and Bear 2004; Whitlock et al. 2006). We induced and followed up the LTP over several days in awake freely moving mutant and wild-type mice. Evoked responses were monitored in the perforant path recorded in the dentate gyrus, an important pathway for hippocampal memory, before and after LTP induction. Following 2 d of baseline recording, tetanic stimulation of the perforant path induced significant potentiation of the population spike for multiple days in both groups (vs. baselines on day 0; *P* < 0.001). However, hippocampal LTP was impaired over 5 d in the mutant mice compared with wild-type littermates (Fig. 4E,F). We also performed electroencephalogram (EEG) recordings in these mice and found that EEG recordings from the somatosensory cortices of these mice displayed abnormalities as measured by the increase in the number of abnormal spikes (Fig. 4G,H). These data align with the clinical features of seizures observed in the individual carrying the G118E mutation as well as in other RTT patients (Glaze et al. 2010). Overall, these data indicate that *Mecp2*^*G118E/y*^ mice recapitulate physiological deficits seen in RTT patients.

### The G118E mutation alters MeCP2 dynamics at chromocenters in live neurons

**Figure 5.**
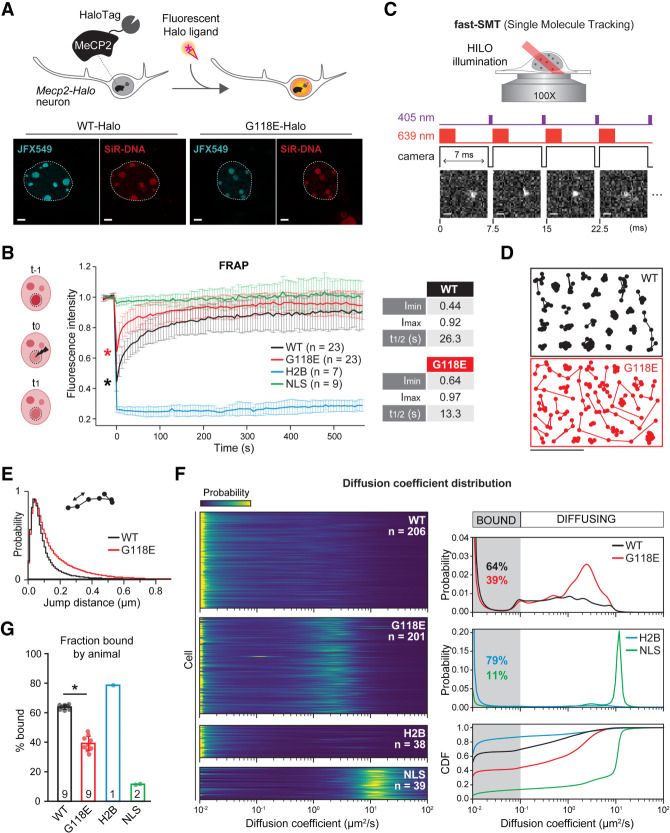
The G118E mutation alters MeCP2-Halo nuclear dynamics in primary cortical neurons. (*A*, *top*) Labeling schematic of Halo-tagged MeCP2 proteins with cell-permeable covalent Halo ligands conjugated to fluorophores for imaging. (*Bottom*) Neurons dissected from *Mecp2*^*WT/y*^ (WT-Halo) and *Mecp2*^*G118E/y*^ (G118E-Halo) show localization of the MeCP2 protein (stained with JFX549; cyan) at DNA-dense heterochromatic foci (stained with SiR-DNA; red). Images are maximum intensity projections of *Z*-stacks acquired from live cells on a laser-scanning superresolution microscope. Scale bar, 2 μm. (*B*) 3D FRAP experiments. (*Left*) Experimental setup. After five frames (*t*_−5_ to *t*_−1_), a selected chromocenter was bleached to approximately half of its fluorescence intensity (between *t*_−1_ and *t*_0_), and the signal recovery was measured at each subsequent frame. (*Right*) WT-MeCP2-Halo (black) and G118E-MeCP2-Halo (red) samples differ in their bleaching depth (asterisks). The HaloTag alone (NLS; green) is so mobile that it hardly gets bleached and recovers immediately. The very stably bound H2B-Halo (blue) shows no substantial recovery within the chosen acquisition time. FRAP is plotted as the mean fluorescence recovery after background subtraction, normalization to the initial fluorescence intensity (set to 1), and acquisition of photobleaching correction. Bars are standard deviations from the mean. *n* indicates the number of neurons used for the analysis. (*) *P* < 0.0001, Mann–Whitney *U*-test (WT vs. G118E, less). The table at the *right* shows WT and G118E minimal fluorescence intensity after bleaching (bleach depth; *I*_min_), maximal fluorescence recovery (*I*_max_), and time to half-recovery (*t*_1/2_, in seconds). (*C*) Fast-SMT experiment setup. (*Top*) Schematic of highly inclined and laminated optical sheet illumination (HILO). (*Bottom*) Illumination and camera sequence with a representative MeCP2-Halo molecule (stained with PA-JF646) detected in four consecutive frames. A short pulse (∼447 μsec) of a low-intensity 405-nm laser photoactivated single PA-JF646 dye molecules during the camera dead time, followed by excitation by a 2-msec pulse of a high-intensity 639-nm laser. Scale bar, 1 μm. (*D*) Randomly sampled trajectories of WT-MeCP2-Halo (red) and G118E-MeCP2-Halo single molecules generated by connecting detections (dots) appearing in consecutive frames of fast-SMT movies. Scale bar, 1 μm. (*E*) Distribution of jump lengths of WT-MeCP2-Halo (black line; *n* = 946,622 jumps) and G118E-MeCP2-Halo (red line; *n* = 813,665 jumps) molecules (see also Supplemental Fig. S6D). (*F*) SASPT analysis of fast-SMT data. (*Left*) Heat map of the marginalized posterior likelihood of diffusion coefficients based on a model of regular Brownian motion with localization error (colors ranging from blue to yellow indicate increasing likelihood). Each row on the *Y*-axis is a cell; *n* specifies the total number of cells analyzed per each group. (*Right*) Averaged distribution of molecules as a function of their diffusion coefficient (posterior occupation) measured in neurons expressing WT-MeCP2-Halo (WT; black), G118E-MeCP2-Halo (G118E; red), H2B-Halo (H2B; blue), and Halo-NLS (NLS; green). The cumulative distribution function (CDF) of the same values highlights differences in the bound fraction (percentage bound; diffusion coefficient <0.1 μm^2^/sec; gray) between the four groups. (*G*) Bound fraction of WT-MeCP2-Halo (WT; black), G118E-MeCP2-Halo (G118E; red), H2B-Halo (H2B; blue), and Halo-NLS (NLS; green) molecules broken down by animal (the number inside each bar indicates how many animals neurons were dissected from). (*) *P* < 0.001, Mann–Whitney *U*-test (WT vs. G118E, greater).

After confirming that G118E is a pathogenic mutation that recapitulates RTT in a mouse model, we next sought to determine how this mutation impairs MeCP2 DNA binding ability in live neurons. MeCP2 nuclear mobility has been linked to its DNA binding ability by SMT experiments performed on mice with *Mecp2* alleles endogenously tagged with the self-labeling HaloTag (Piccolo et al. 2019). To explore the impact of the G118E mutation on MeCP2's dynamic behavior, we generated a knock-in mouse model carrying the G118E mutation on the endogenously Halo-tagged *Mecp2* allele. Western blot and immunostaining confirmed that the G118E mutation similarly reduced the protein level of Halo-tagged MeCP2 compared with wild-type (WT) MeCP2-Halo protein in the mouse brain, as expected (Supplemental Fig. S5). We then isolated cortical neurons from *Mecp2-Halo*^*WT/y*^ and *Mecp2-Halo*^*G118E/y*^ male pups, matured them in culture for 12–15 d, and stained them with cell-permeable covalent Halo ligands conjugated to JaneliaFluor dyes (Fig. 5A, top; Supplemental Fig. S6A**)**. We first confirmed by live-cell superresolution imaging that both WT-MeCP2-Halo and G118E-MeCP2-Halo proteins correctly localize to condensed heterochromatic foci (chromocenters) by dual JFX549 and SiRDNA staining (Fig. 5A, bottom; Supplemental Fig. S6B, left). We next measured global MeCP2 nuclear dynamics at these chromocenters using fluorescence recovery after photobleaching (FRAP). We bleached entire chromocenters to ∼40%–50% of their initial fluorescence intensity and measured their fluorescence recovery over time (Fig. 5B; Supplemental Fig. S7A), using volumetric imaging (Lemcke et al. 2016) with a frame rate of ∼6 sec to account for the substantial movement of heterochromatic foci during the acquisition time (see the Materials and Methods; Supplemental Fig. S7B,C). Despite the G118E mutation resulting in overall reduced MeCP2 levels (Figs. 1B,C, 2B; Supplemental Fig. S5), we were technically constrained to select WT-MeCP2-Halo and G118E-MeCP2-Halo neurons and chromocenters with similar absolute fluorescence intensities for reliable FRAP measurements (see the Materials and Methods; Supplemental Fig. S7D**)**. As a reference for a fast-recovering protein and a slow-recovering one, we measured FRAP dynamics of a HaloTag alone fused to a nuclear localization signal (Halo-NLS) and a Halo-tagged histone 2B (H2B-Halo) stably introduced in wild-type cortical neurons via lentiviral vectors (Supplemental Fig. S6B, right**)**. Halo-NLS and H2B-Halo controls behaved as expected, with NLS recovering immediately and H2B recovering only minimally (Fig. 5B; Supplemental Fig. S7A,E**)**. In neurons carrying the G118E mutation, the bleaching depth was significantly reduced (asterisks in Fig. 5B), suggesting that dark, mobile molecules promptly diffused out of the bleached area to be replaced by fluorescent ones within the few seconds intervening between photobleaching and the next acquired frame (*t*_0_ in Fig. 5B). This observation suggests that more and faster-diffusing G118E-MeCP2-Halo proteins are readily available for exchange compared with WT-MeCP2-Halo proteins. Given our slow frame rate (∼6 sec), we can safely assume that diffusing molecules mostly affected the bleaching depth, while the measured fluorescence recovery largely derived from bound molecules dissociating from and leaving the bleached chromocenter. Chromocenters in both WT-Halo and G118E-Halo neurons recovered >90% of their initial fluorescence, with mutant cells reaching almost full recovery (97% vs. 92% in WT cells) (Fig. 5B; Supplemental Fig. S7E). Notably, the half-time to recovery was twice as fast in G1118E-MeCP2-Halo neurons than in WT neurons (13 sec vs. 26 sec, respectively), pointing to a more dynamic binding of G118E molecules compared with WT ones (Fig. 5B). While we refrained from fitting FRAP recovery curves to any kinetic model to estimate MeCP2 dwell times on chromatin due to the complex nature of chromocenters (see the Materials and Methods), both the reduced bleach depth and the faster half-time to recovery of G118E neurons indicate that chromocenters of mutant neurons exchange MeCP2 proteins with the rest of the nucleus faster than wild-type cells.

### Single G118E MeCP2 molecules have impaired chromatin binding in live neurons

Having assessed the effect of the G118E mutation on MeCP2 dynamics at chromocenters, we next investigated how the mutation affects the binding and diffusing behavior of single MeCP2 molecules in real time at a single-cell level by fast-SMT. We optimized a sequential labeling strategy with a photoactivatable (PA) JF646 dye (for single-molecule detection) followed by the JFX549 dye (for nuclear masking) to track single MeCP2-Halo molecules in the nucleus of live cortical neurons with fast-SMT (see the Materials and Methods). We sampled ∼20 cells per pup using nine *Mecp2-Halo*^*WT/y*^ and nine *Mecp2- Halo*^*G118E/y*^ pups, for a total of 409 cells. In fast-SMT, highly inclined and laminated optical sheet illumination (HILO) (Tokunaga et al. 2008) coupled with stroboscopic excitation pulses at high power and fast acquisition times (7 msec) minimizes motion blur and allows detection of both bound and fast-diffusing molecules with high signal to noise ratio (Fig. 5C). After nuclear masking in the JFX549 channel to remove cytoplasmic signal (Supplemental Fig. S6C), we connected single molecules appearing in consecutive frames into hundreds of thousands of trajectories using the Quot tracking algorithm (see the Materials and Methods) and immediately detected a higher mobility of the G118E-MeCP2-Halo protein compared with WT (Fig. 5D). Indeed, plotting the distribution of the jump lengths (i.e., the distance covered by each molecule between consecutive frames) with Spot-On (Hansen et al. 2018), we confirmed that G118E-MeCP2-Halo molecules consistently travel longer distances than WT-MeCP2-Halo molecules and thus are more diffusive (Fig. 5E). As a control, fast-SMT on Halo-NLS and H2B-Halo neurons gave us the opposing jump length distributions expected for a very mobile and an “immobile” protein, respectively (Supplemental Fig. S6D). We next analyzed SMT trajectories with the SASPT package (Heckert et al. 2022), a Bayesian-based approach that infers the distribution of a protein's diffusion coefficient without a priori assumptions about the underlying number of states (e.g., binding or slow or fast diffusing alone or in association with other partners). SASPT unequivocally established that the G118E mutation impairs MeCP2-Halo association with chromatin: On average, at any given time, only ∼39% of G118E-MeCP2-Halo molecules were “bound” (diffusion coefficient <0.1 μm^2^/sec) compared with ∼64% of WT-MeCP2-Halo proteins, resulting in the emergence of a population of G118E molecules with a distinct diffusion coefficient of ∼2–3 μm^2^/sec (Fig. 5F). This is substantially slower than free Halo protein (NLS; >10 μm^2^/sec), and only full-length WT-MeCP2-Halo or G118E-MeCP2-Halo proteins were detected by Western blot (Supplemental Fig. S5A), implying that this diffusing population does not consist of degraded protein fragments. As an additional technical control, the majority (79%) of H2B-Halo molecules bound chromatin in neurons, while only 11% of Halo-only proteins were immobile (Fig. 5F). G118E-MeCP2-Halo had a lower bound fraction across all animals (Fig. 5G; Supplemental Fig. S6E), and results were consistent in different neurons from each animal, albeit with greater cell-to-cell variability for G118E-MeCP2-Halo than for WT-MeCP2-Halo (Supplemental Fig. S6F).

Taken together, FRAP and fast-SMT experiments in living neurons indicate that G118E MeCP2 is less bound and more mobile than WT MeCP2, which speeds up protein exchange between chromocenters and the rest of the nucleus.

### The G118E mutation impairs MeCP2 chromatin binding independent of reduced protein levels

**Figure 6.**
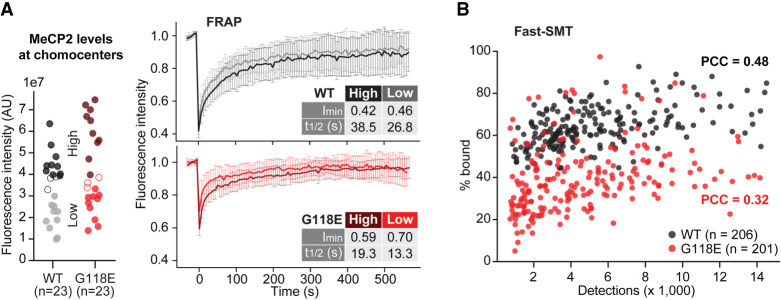
The G118E mutation alters MeCP2 nuclear dynamics independently of reduced protein levels. (*A*) Fluorescence recovery curves for WT (black) and G118E (red) neurons expressing high (top 10 expressing cells; dark shade) or low (bottom 10 expressing cells; light shade) MeCP2 levels at the targeted chromocenters. Details on absolute intensity measurements are shown in Supplemental Figure S7D. FRAP is plotted as in Figure 5B. See Supplemental Figure S8B for additional recovery values. (*B*) Scatter plot correlating the number of single MeCP2-Halo molecules (stained with the photoactivatable JF-PA646 dye) detected per cell in fast-SMT experiments (a proxy for protein levels; *X*-axis) with the fraction of molecules bound to chromatin (percentage bound; *Y*-axis). Each dot is a single imaged neuron, either wild type (black) or carrying the G118E mutation (red) in *Mecp2*. *n* = total neurons imaged per conditions. (PCC): Pearson's correlation coefficient.

Several described MBD point mutations result in decreased MeCP2 protein levels, at least in some cases ascribed to reduced protein stability, and G118E is no exception (Fig. 2B,C; Goffin et al. 2012; Johnson et al. 2017; Lamonica et al. 2017; Gandaglia et al. 2019). We thus asked whether the binding impairment that we observed in live neurons is an intrinsic defect of the G118E mutant or a simple consequence of its reduced protein levels. Because both our FRAP and fast-SMT measurements contain information on MeCP2 levels in single cells, we could correlate MeCP2's nuclear dynamics with its protein amounts. Starting with FRAP data, for both WT and G118E neurons, we grouped cells into high and low expressing based on MeCP2 levels at targeted chromocenters and contrasted them (Fig. 6A). The analysis showed a minimal effect of protein concentration on fluorescence recovery, with G118E neurons that express the highest MeCP2 levels still having faster dynamics than WT neurons that express the lowest protein levels (Fig. 6A; Supplemental Fig. S8A). To confirm this observation in the fast-SMT data set, we used the number of single molecules detected in each movie as a proxy for protein levels in single cells, since the number of photoactivated (JF-PA646-stained) MeCP2-Halo molecules in any given neuron was proportional to the total number of MePC2 molecules present in that cell. We then correlated the total number of detections in any given cell with the fraction bound as inferred by SA-SPT for the same cell. In WT neurons, such analysis revealed only a moderate correlation (Pearson's correlation coefficient [PCC] = 0.48) (Fig. 6B). Such correlation was modest yet robust, since it disappeared when we randomly paired values (PCC = 0.01) and was much higher than in the H2B-Halo and Halo-NLS data sets (Supplemental Fig. S8B,C). In G118E neurons, the correlation was milder than in WT cells (PCC = 0.3, vs. PCC = −0.03 in randomly paired values), indicating that the reduction in fraction bound is largely independent of total protein levels (Fig. 6B; Supplemental Fig. S8C). While overall we detected fewer MeCP2 molecules in G118E cells compared with WT ones (consistent with the mutation globally reducing protein levels) (Supplemental Fig. S8D), G118E neurons with numbers of detection similar to those of WT neurons still displayed an obvious binding defect (Fig. 6B).

In summary, both FRAP and fast-SMT data demonstrate that the DNA association defect is intrinsic to the G118E mutant protein. Moreover, single-cell imaging experiments allowed us to appreciate that G118E cells with MeCP2 levels comparable with WT cells still display altered MeCP2 nuclear dynamics. These results complement and corroborate ChIP-qPCR and CUT&RUN experiments (Fig. 2D–F), demonstrating that the reduced binding of G118E-MeCP2 is not a mere consequence of its diminished protein levels.

## Discussion

In this work, we characterize a novel mutation in *MECP2* (c.353G>A, p.Gly118Glu [G118E]) that was identified in a young boy. Quantitative measurement of MeCP2 protein in neurons derived from his fibroblasts showed a 40% reduction in protein levels. To further understand the pathogenicity of the mutation, we generated and comprehensively characterized a G118E knock-in mouse model with or without a self-labeling HaloTag at the *Mecp2* locus, which confirmed that this mutation reduces MeCP2 protein levels and partially impairs DNA binding. The G118E mutation leads to behavioral and physiological deficits in mice, recapitulating all the symptoms seen in the affected individual. Importantly, single-molecule imaging in live cells revealed that the G118E mutation reduces MeCP2's association with chromatin independently of protein levels, providing new insight into how some MBD mutations in MeCP2 might affect its interaction with chromatin.

Most studies so far have focused on studying male mice carrying severe RTT-causing loss-of-function mutations (null or abolishing DNA binding), which occur predominantly in female patients. Male mice carrying mild pathogenic mutations identified in male patients (e.g., the G118E mouse model developed in the current study) are an important disease model to assess functional consequences of any particular mutation in the absence of mosaicism. Moreover, it is worth noting that ∼75% of the RTT-causing mutations are not null alleles but retain partial function of the protein and some binding to the DNA. Developing a model for RTT that is not as severe allows for testing therapeutic strategies that could be applied to other RTT-causing alleles with decreased protein level or changes in DNA binding ability. On the milder end, the A140V mutation in males causes intellectual disability and neuropsychiatric symptoms rather than classic Rett syndrome (Jentarra et al. 2010), and mice carrying the A140V mutation also display no obvious life span or physiological phenotypes (Jentarra et al. 2010). Unlike A140V mice, the G118E mouse model recapitulated all the phenotypes observed in RTT patients by 8–9 wk of age, making it an optimal model of RTT with mutations that do not abolish protein function. More importantly, single-molecule imaging in living neurons detected a robust reduction of the fraction of molecules bound to chromatin and a corresponding increase in diffusing of unbound G118E molecules. These alterations were not previously observed in R133C, another mild mutation that retains partial DNA binding of MeCP2, but were observed in the severe R106W mutation that completely abolishes MeCP2's DNA binding (Piccolo et al. 2019). Therefore, our study demonstrates that live-cell imaging can discriminate MeCP2 with compromised DNA binding in a milder disease model in a native cellular environment, which could directly be used to screen and evaluate therapeutic interventions targeting pathological proteins.

Single-cell imaging measurements also allowed us to uncouple the effects of the G118E mutation on MeCP2 protein levels from its DNA binding defect. While this has been achieved for other MeCP2 mutations by in vitro binding assays with recombinant proteins (Nikitina et al. 2007; Baker et al. 2013; Heckman et al. 2014; Zhang et al. 2022b), in vivo measurements with full-length proteins expressed at endogenous levels in their native chromatin environment were never attempted. Correlating the fraction of MeCP2 molecules engaged in chromatin binding with protein levels in single neurons revealed that the G118E mutant protein is intrinsically defective in chromatin engagement: Mutant neurons with equal or even higher levels than wild-type cells still display altered DNA binding. Similar conclusions came from FRAP measurements of chromocenter dynamics. While endogenous protein levels did not rescue the G118E binding defect, we did observe a moderate correlation between MeCP2 concentration and fraction bound (not observed for Halo-NLS or for H2B-Halo), as well as a mild effect of initial protein levels at chromocenters on their fluorescence recovery after photobleaching (Fig. 6; Supplemental Fig. S8B). Such an effect is consistent with the documented ability of MeCP2 to self-interact (Georgel et al. 2003; Wang et al. 2020; Zhang et al. 2022a): At increasing protein concentrations, MeCP2 molecules already bound to DNA might facilitate chromatin docking of diffusing ones.

Heterochromatic foci are densely methylated, so FRAP measurements are likely a combination of MeCP2 molecules engaging with a single locus and molecules jumping from one methylated site to the next before leaving the bleached chromocenter. Because of this complexity, we did not apply any kinetic modeling procedures to the FRAP results, and whether G118E molecules reside on chromatin long enough to carry out their function remains to be determined, possibly by significantly increasing the level of the mutant protein. Given the intrinsic defect in chromatin engagement of the G118E MeCP2 protein (40% drop in fraction bound as estimated by fast-SMT), we predict that the mutant protein will need to be overexpressed ∼1.5-fold above WT levels to achieve a comparable total number of bound molecules. Our prediction agrees well with functional rescues of other MeCP2 MBD mutations that required twice as much mutant MeCP2 to normalize some of the RTT phenotypes (Lamonica et al. 2017).

How does the G118E mutation affect MeCP2 engagement with chromatin? Crystal structures of MeCP2 MBD domain in complex with methylated DNA position glycine 118 at the C-terminal end of a loop that forms hydrogen bonds with the methylated base through arginine 111 (Ho et al. 2008). It is tempting to speculate that the G118E substitution alters the loop structure, interfering with MeCP2 docking to DNA. Homology modeling of the WT and G118E mutated loops with the Rosetta LoopModeler protocol predicted a substantial conformational change for the G118E mutation (Supplemental Fig. S8E). The change likely resulted from the reduced propensity of glutamate to adopt the backbone φ and ψ angles that are required by the native glycine to adopt the crystallographic conformation. Although the mutant maintains key hydrogen bonds with the DNA through arginine 111, consistent with its ability to bind DNA, the conformational change observed in the simulation illustrates a preference of the WT protein to pack against the DNA that is significantly diminished in the mutant. Whether this prediction reflects an actual conformation adopted in vivo by the G118E mutant will need to be experimentally validated.

An alternative explanation for lower protein levels and higher fraction of fast-diffusing G118E molecules is that the mutation results in an unstable protein that is progressively degraded and/or misfolded and thus diffuses faster than the wild-type one. Although we cannot exclude such a hypothesis, a few observations tend to discredit it. First, we did not see predominant degradation products by Western blot analysis of brain lysates probed with either an anti-Halo antibody or an MeCP2 antibody (Supplemental Fig. S5A). Second, the diffusion coefficient of G118E molecules (2–3 µm^2^/sec) was still significantly slower than free Halo proteins (>10 µm^2^/sec) and fell within the broad diffusion range of WT molecules (0.1–9 µm^2^/sec), suggesting that we were looking at an intact protein. Finally, if any misfolding happened, it must have spared the Halo moiety, since the tag integrity was required for proper labeling and detection. In fact, the conformation change predicted by the Rosetta homology modeling discussed above was pronounced but restricted to the protein loop that contacted methylated DNA, which was very unlikely to cause a shift in MeCP2's diffusive behavior (Supplemental Fig. S8E).

In conclusion, we generated two orthogonal models and established an in-depth pipeline to molecularly characterize new and rare mutations in *MECP2* to determine their pathogenicity. Remarkably, single-molecule imaging experiments in live cells provided new insight into how G118E mutation alters MeCP2's protein dynamics and function in real time. Our findings, together with other recent studies from our and other laboratories (Liu et al. 2019; Parolia et al. 2019; Piccolo et al. 2019; Chen et al. 2022), consolidate the notion that live-cell single-molecule imaging is a powerful new tool to discriminate physiological from pathophysiological protein dynamics in a native cellular environment. Given that the G118E mutation leads to similar changes in MeCP2 abundance in both mouse brains and patient-derived iNeurons, the next important question is whether the G118E mutation also affects MeCP2's chromatin association dynamics in human neurons, which would be a more disease-relevant platform for drug discovery. Ultimately, the combination of our live-cell imaging tool with newly established disease models provides a platform to directly screen and evaluate new therapeutic interventions that can normalize the DNA binding capacity of pathogenic MeCP2 protein. Combined with CRISPR/Cas9 genome-editing technologies and the accessibility and versatility of hiPSC-based disease models, our approach is highly transferable to other pathological settings.

## Materials and methods

### Generation of *Mecp2*^*G118E/y*^ iPSCs

To initiate reprogramming, primary human male dermal fibroblasts harboring the *MECP2* G118E mutation were infected with nonintegrating Sendai viruses expressing OCT4, SOX2, KLF4, and C-MYC (CytoTune-iPS 2.0; Thermo Fisher). Clonal human induced pluripotent cell (hiPSC) colonies were manually picked and expanded under feeder-free conditions using hESC-qualified Matrigel (Corning) and mTeSR1 medium (Stem Cell Technologies). HSCC-103iPS clone 15 was shown to have a normal male karyotype at passage 7 and subsequently was used for genome editing.

### Genome editing of *Mecp2*^*G118E/y*^ iPSCs

*Mecp2*^*G118E/y*^ clone 15 iPSCs were adapted to single-cell passaging using Accutase and StemFlex medium (Thermo Fisher Scientific) prior to nucleofection. On the day of nucleofection, iPSCs were pretreated with 10 µM Y-27632 for 30 min. Cells were dissociated to single cells using Accutase, and 300,000 cells were nucleofected with 200 pmol of sgRNA (Synthego) complexed to 40 pmol of Cas9 protein (TrueCut Cas9 protein v2; Thermo Fisher Scientific) and 50 pmol of a single-stranded DNA repair template using the P3 primary cell 4D-nucleofector X kit S (Lonza program CA-137). Nucleofected cells were seeded onto a Matrigel-coated 10-cm plate in StemFlex medium supplemented with 10 µM Y-27632 for the first 24 h. Colonies were manually picked and screened by genomic PCR followed by restriction enzyme digestion with HhaI.

### Neuron differentiation: NGN2

The iPSCs were plated in 12-well plates and first infected with lentivirus (Addgene 66810) packaged with rtTA with hygromycin selection and then infected with lentivirus packaged with NGN2-EGFP with blasticidin selection (Crutcher et al. 2019). For biochemistry assays, the cells were plated in 24-well plates and induced into the neurons using doxycycline and neural induction medium (DMEM/F12:neurobasal [1:1] supplemented with 1× B27, 1× N2, 2 nM GlutaMax) for 4 d and neural differentiation medium (neuobasal with 1× B27, 2 mM GlutaMax, 20 ng/mL BDNF, 10 ng/mL GDNF, 10 ng/mL NT-3, 100 μM db-cAMP, 200 μM ascorbic acid) afterward. The neurons were harvested 60 d after differentiation.

### Neuron differentiation: NPCs

Differentiation and culturing of neural progenitor cells from human patient-derived induced pluripotent stem cells was performed following our earlier published protocol (Jiang et al. 2017; Rousseaux et al. 2018a,b; Vázquez-Vélez et al. 2020).

### Mouse generation

#### Animals

Mice were housed in an AAALAS-certified level 3 facility on a 14-h light cycle. The mice were weaned at 21 d after birth and housed with three to four littermates per cage. G118E mice were generated at Baylor College of Medicine (see below), backcrossed at least five generations, and maintained in C57B6/J background. F1 hybrids of C57B6/J and 129S1 were used for behavioral and physiology tests. The male offspring were used for analysis. The experimenter was blinded to the genotypes while performing all behavior assays. Other experiments were performed without blinding to genotypes. All procedures to maintain and use the mice were approved by the Institutional Animal Care and Use Committee for Baylor College of Medicine and Affiliates.

#### Generation of G118E knock*-*in and Mecp2^G118E^*-*halo mice

The G118E mutation was introduced endogenously to wild-type and previously established *Mecp2-Halo* mice (Piccolo et al. 2019) on a pure C57Bl/6J background via CRISPR/Cas9-mediated gene editing. Briefly, one sgRNA targeting the mutation site was designed from Benchling and synthesized by IDT. The single-strand ODNs were synthesized by GenScript. Silent mutations were made for easy genotyping: gRNA sequence (CATCATACTTTCCAGCAGAT) and ssODN sequence (CTCGGCTTCCCCCAAACAGCGGCGCTCCATTATCCGTGACCGGGGACCTATGTATGATGACCCCACCTTGCCTGAAGGTTGGACACGAAAGCTTAAACAACGCAAAAGCGGAAGGAGCGCCGAAAAGTATGATGTATATTTGATCAAGTAAGTAAGAGCAAGTCTTGTGTCTATAGAACAAGA).

Next, gRNA was in vitro transcribed with the MEGAshortscript T7 transcription kit (Invitrogen). On the day of injection, Cas9 protein (PNA Bio) and sgRNA with ssODNs were injected (pronuclear) into ova from C57Bl/6 female mice and transferred into oviducts of pseudopregnant females. The following primers were used to distinguish the G118E mice: forward: GCCACTACAACCTTCAGCCCACCAT and reverse: TCGGCGCTCCTTCCGCTTTTGCG.

*Mecp2-Halo* and *Mecp2^G118E^-Halo* mice were transferred to the University of California, Berkeley, where they were mated and used for imaging in accordance with the recommendations in the guide for the care and use of laboratory animals of the National Institutes of Health and following the animal use protocol (AUP-2015-09-7988-2) approved by the Animal Care and Use Committee (ACUC) of the University of California, Berkeley. *Mecp2-Halo*^*WT*^/^*G118E*^ heterozygous females were mated with *Mecp2*-*Halo*^*WT/y*^ hemizygous males to generate both *Mecp2-Halo*^*WT/y*^ and *Mecp2-Halo*^*G118E/y*^ male pups in the same litter. Newborn male pups were distinguished from females by their characteristic pigment spot on the scrotum (Wolterink-Donselaar et al. 2009) and selected for dissection and imaging. Pups’ male gender and the presence or absence of the G118E mutation were assessed by PCR with the following primers: y-specific forward (TGGGACTGGTGACAATTGTC), y-specific reverse (GAGTACAGGTGTGCAGCTCT), G118E-specific forward (CGCAAAAGCGGAAGGAGC), G118E-specific reverse (CCAGACCTAATCCACCACCA), WT-specific forward (AGGAAGTCTGGCCGATCTG), and WT-specific reverse (TGCTCAGAAGCCAAAACAGC). Wild-type C57Bl/6J mice were also mated to perform lentiviral transduction of wild-type neurons with Halo-NLS and H2B-Halo constructs, which served as an imaging control (details below).

### Brain lysate preparation and Western blot

Brains were dissected and homogenized in cold lysis buffer (20 mM Tris-HCl at pH 8.0, 180 mM NaCl, 0.5% NP-40, 1 mM EDTA, 2% SDS, Complete protease inhibitor [Roche]). Lysates were sonicated for 10 min with Biorupter Pico (Diagenode), rotated for 20 min at room temperature, and then centrifugated at maximum speed for 20 min. The supernatant was then mixed with NuPAGE sample and reducing buffer, heated for 10 min at 95°C, and run on a NuPAGE 4%–12% Bis-Tris gradient gel with MES SDS running buffer (NuPAGE). Separated proteins were transferred to nitrocellulose membrane using NuPAGE transfer buffer for 2.5 h at 4°C. The membrane was blocked with 5% BSA in TBS with 2% Tween-20 (TBST) for 1 h and incubated with primary antibody overnight at 4°C. After three washes with TBST, the membrane was incubated with secondary antibody in 0.5× Odyssey blocking buffer for 1–2 h at room temperature followed by washing. Images were acquired on a LiCOR CLx imager. Antibodies used were anti-MeCP2 (D4F3; 1:1000; Cell Signaling Technologies 3456), anti-HaloTag (1:1000; Promega G9211), mouse anti-GAPDH (1:10,000; Abcam ab8245), and rabbit anti-PCNA (Proteintech 10205-2-AP).

### Immunofluorescence

Animals were anaesthetized with a mix of 37.6 mg mL^−1^ ketamine, 1.92 mg mL^−1^ xylazine, and 0.38 mg ml^−1^ acepromazine and transcardially perfused with 20 mL of PBS followed by 100 mL of cold PBS-buffered 4% paraformaldehyde (PFA). The brains were removed and postfixed overnight in 4% PFA. Next, the brains were cryoprotected in 4% PFA with 30% sucrose for two additional days at 4°C and embedded in optimum cutting temperature (O.C.T.; Tissue-Tek). Free-floating 40-μm brain sections were cut using a Leica CM3050 cryostat and collected in PBS. The sections were blocked for 1 h in 2% normal goat serum and 0.3% Triton X-100 in PBS at room temperature. Sections were then incubated overnight at 4°C with rabbit anti-MeCP2 antibody (1:1000; Cell Signaling 3456). The sections were washed three times for 10 min with PBS and incubated for 2 h at room temperature with goat antirabbit antibody (Alexa fluor 488; 1:500; Invitrogen A-11034). For MeCP2-Halo detection, sections were incubated with HaloTag ligand TMR (Promega G8252) without secondary antibody incubation. Sections were washed three times for 10 min with PBS and mounted onto glass slides with VectaShield mounting medium with DAPI (Vector Laboratories).

### Gene expression analysis by qRT-PCR

Total RNA was extracted from different brain regions of adult mice using a Qiagen RNeasy mini kit, and 3 μg was used to synthesize cDNA by M-MLV reverse transcriptase (Life Technologies). qPCR was performed in a CFX96 real-time system (Bio-Rad) using PerfeCTa SYBR Green fast mix (Quanta Biosciences). Sense and antisense primers were selected to be located on different exons and the RNA was treated with DNase to avoid false-positive results caused by DNA contamination. The specificity of the amplification products was verified by melting curve analysis. All qPCR reactions were conducted in technical triplicates, and the results were averaged for each sample, normalized to GAPDH levels, and analyzed using the comparative ΔΔCt method. The following primers were used in the RT–qPCR reactions: *MECP2* (common to humans and mice; forward: 5′-TATTTGATCAATCCCCAGGG-3′ and reverse: 5′-CTCCCTCTCCCAGTTACCGT-3′), *Gapdh* (mouse-specific; forward: 5′-GGCATTGCTCTCAATGACAA-3′ and reverse: 5′-CCCTGTTGCTGTAGCCGTAT-3′), and *GAPDH* (human-specific; forward: 5′-AAAGGGTCATCATCTCCGCC-3′ and reverse: 5′-TCATGGATGACCTTGGCCAG-3′).

### Chromatin immunoprecipitation

ChIP was performed as previously described (Ito-Ishida et al. 2018) with minor modifications. Frozen tissue was cross-linked by incubating in 1% PFA (Fisher 28906) in PBS for 10 min at room temperature. The fixation was quenched with 125 mM glycine in ice-cold PBS, and then the samples were homogenized in hypotonic solution (10 mM Tris at pH 7.5, 0.5% Igepal CA-630, 10 mM NaCl, 30 mM MgCl_2_, 1:1000 RNase cocktail [Ambion 2286], 1 mM PMSF, 1× CPI). Nuclei were collected at 3000 rpm in a tabletop centrifuge and resuspended in nucleus lysis buffer containing 50 mM Tris (pH 8), 10 mM EDTA, 1% SDS, 1 mM PMSF, and 1× CPI. The sample was incubated in the nucleus lysis buffer for 15 min on ice and sonicated by Bioruptor Pico (Diagenode) for 10 cycles (30 sec off/ 30 sec on) to obtain DNA fragments of 100–500 bp. The chromatin samples were flash-frozen in liquid nitrogen and stored at −80°C until needed. To perform ChIP, chromatin samples were first diluted based on their DNA concentration, further diluted in ChIP dilution buffer (0.01% SDS, 1.1% Triton-X, 1.2 mM EDTA, 16.7 mM Tris at pH 8.1, 167 mM NaCl), and precleared with Protein A Dynabeads (Invitrogen) for 1 h. Approximately 5 μg of chromatin was incubated overnight at 4°C with anti-MeCP2 antibody per the manufacturer's recommendation (Abcam ab2828). An aliquot of 10% of the precipitated chromatin was stored as input. The next day, 40 μL of Protein A Dynabeads was added, and the sample was rotated for 3 h at 4°C. The beads were then washed in 700 μL each of low-salt buffer (0.1% SDS, 1% Triton-X, 2 mM EDTA, 20 mM Tris at pH 8.1, 150 mM NaCl), high-salt buffer (0.1% SDS, 1% Triton-X, 2 mM EDTA, 20 mM Tris at pH 8.1, 500 mM NaCl), and LiCl wash buffer (250 mM LiCl, 1% Igepal-CA630, 1 mM EDTA, 10 mM Tris at pH 8.1) and twice with TE + NaCl buffer (10 mM Tris HCl, 1 mM EDTA, 50 mM NaCl) for 5 min each at room temperature. The beads were eluted twice in 250 μL of elution buffer (1% SDS, 100 mM NaHCO_3_) for 15 min each at 65°C. Precipitated chromatin and input samples were reverse-cross-linked and treated with proteinase K. ChIP DNA and input DNA were recovered using a PCR purification kit (Qiagen) and used for qPCR. The following primers were used for ChIP-qPCR experiments: *Major satellite repeat* (forward 5′-CATCCACTTGACGACTTGAAAA-3′, reverse 5′-GAGGTCCTTCAGTGTGCATTT-3′) and *Bdnf* (forward 5′-GGATTCCCTTCATCCTCAGAT-3′, reverse 5′-CCAAAGAGTAAGTGTGCCCTTC-3′). Enrichment over input (percentage) was plotted in graphs. Statistical analyses were performed using ΔCt values.

### CUT&RUN

CUT&RUN (cleavage under targets and release using nuclease) was performed as published previously (Skene and Henikoff 2017; Bajikar et al. 2022) with a few modifications. See the Supplemental Material for a detailed description.

### Isolation of primary cortical neurons

Mouse primary neurons were isolated and cultured as previously described (Zhou et al. 2022) with minor modifications. P0–P2 *Mecp2-Halo*^*WT/y*^ and *Mecp2-Halo*^*G118E/y*^ males were sacrificed by decapitation, and cortices were dissected from brains under a stereo microscope (AmScope SM-1TSZZ-144S-10M) with sterile surgical tools in ice-cold Hank's balanced salt solution with no Ca^2+^ or Mg^2+^ (Invitrogen 14110-172) added and 5 mL/L penicillin/streptomycin antibiotics (Invitrogen 15140-122). Meninges were removed from cortices, and the dissected tissue was transferred to 500 μL of equilibrated complete neurobasal media (CNB) composed of neurobasal media (Invitrogen 21103049), 1× B27 supplement (Invitrogen 17504-044), 0.5× GlutaMax (Invitrogen 35050-061), and 5 mL/L penicillin/streptomycin. Neurons were dissociated from cortical tissue with the Papain dissociation system kit, prepared according to the manufacturer's instructions (Worthington LK003153). CNB was carefully removed from the tissue and replaced with 500 μL of pre-equilibrated Papain solution. Cortices were digested in a thermomixer at 1000 rpm for 45 min at 37°C. Papain was replaced with 500 μL of pre-equilibrated inhibitor followed by incubation in a thermomixer at 1000 rpm for 5 min at 37°C. The inhibitor was replaced with 1 mL of equilibrated CNB, and the dissociated tissue was triturated to a single-cell suspension by vigorous pipetting. Cells were passed through a prewetted 40-μm cell strainer to remove cell clumps and counted in Trypan Blue (Sigma T8154-20 ML) with a hemocytometer. Cells were plated in 12-well plates onto 18-mm high-precision microscope cover glasses (no. 1.5H; Marienfeld ES0117580, distributed by Azer), which were plasma-cleaned (Diener electronic Femto plasma system) prior to coating for 1 h at 37°C with a poly-D-lysine solution (2.5 mg/mL final in water; Sigma-Aldrich P6407). After coating, cover glasses were rinsed three times with sterile water and dried in the laminar hood before cells were seeded onto them (seeding density 6 × 10^5^ cells per well). Neurons were allowed to attach to the cover glasses for 2 h in a regular cell culture incubator at 37°C and 5% CO_2_ before the media was gently replaced to remove unattached glial cells. Dissected neurons were matured in culture for 10–15 d prior to staining and imaging, with half of their media changed every 4 d.

### Live*-*cell staining of primary cortical neurons

Halo-fused proteins were stained with cell-permeable HaloTag ligands conjugated to bright JaneliaFluor (JF) dyes with different spectral, biophysical, and chemical properties (all kindly provided by Luke Lavis). All incubations were done in a regular cell culture incubator.

For confocal superresolution imaging, neurons were stained with the JFX549 dye (50 nM in CNB) for 30 min. After two quick washes in CNB, cells were incubated with 500 nM SiR-DNA live-cell nuclear stain (Cytoskeleton CY-SC00) in conditioned CNB for 1 h.

For fast single-molecule tracking (fast-SMT), neurons were sequentially stained with photoactivatable PA-JF646 Halo ligand (25 nM in CNB) to track single molecules, and JFX549 Halo ligand (5 nM in CNB) to create nuclear masks for analysis (see below). Each dye was incubated with cells for 30 min and quickly washed twice before the next incubation. Cells were destained in conditioned CNB for 1 h prior to imaging.

For fluorescence recovery after photobleaching (FRAP) of wild-type and G118E mutant MeCP2 proteins, neurons were simultaneously stained for 30 min with JFX549 and JFX650 (Grimm et al. 2021) dyes (50 nM each in CNB). After two quick washes in CNB, cells were destained in conditioned CNB for 1 h prior to imaging.

All coverslips were mounted in conditioned CNB in an imaging chamber with a stainless steel bottom (ALA Scientific Instruments MS-508S) and subjected to live-cell imaging.

### Lentiviral transduction of primary cortical neurons

Neurons were cultured for 1 wk prior to infection with a lentiviral vector expressing a HaloTag fused to c-Myc nuclear localization signal (NLS) or a C-terminally Halo-tagged H2B driven by an L30 promoter followed by an IRES and an antibiotic resistance gene (puromycin or neomycin) at a multiplicity of infection (MOI) of 1. Media was replaced 24 h after infection. The lentiviral vectors were a pHAGE backbone (Pan et al. 2008) packaged in HEK293T cells by calcium phosphate transfection with the second-generation packaging plasmids pCMVR8.74 (Addgene 22036) and pMD2.G (Addgene 12259) and were titrated in HEK293T cells.

### Live*-*cell imaging of primary cortical neurons

Superresolution imaging and FRAP of live cortical neurons were performed on a Zeiss LSM 900 laser-scanning microscope with an inverted Axio Observer equipped with an Airyscan 2 detector, a motorized stage, and an incubation chamber maintaining the sample at 37°C and 5% CO_2_, operated by Zen 3.1 blue edition software.

Superresolution images of MeCP2-Halo and SiR-DNA staining were acquired using a 63× plan-apochromat NA1.4 oil immersion objective at 8.8× zoom, corresponding to a 44-nm *XY* pixel size. JFX549 was excited with a 561-nm diode laser set at 0.8% with a master gain of 850 V, and SiR-DNA was excited with a 640-nm diode laser set at 0.2% with a master gain of 850 V. The two channels were acquired sequentially. Detection was performed on an Airyscan 2 detector consisting of 32 GaAsP photomultiplier tube detectors with a detector range of 566–630 nm for JFX549 and 645–700 nm for SiR-DNA. For each cell and channel, 27–46 *Z*-planes were acquired with a *Z* interval of 180 nm.

Superresolution images of Halo-NLS, H2B-Halo, and SiR-DNA staining were acquired with a 40× plan-apochromat NA1.3 oil immersion objective at 4× zoom, corresponding to a 78-nm *XY* pixel size. Excitation and detection were as described above for MeCP2-Halo. For each cell, a single plane was acquired. Airyscan deconvolution was performed with Zen 3.1 blue edition software with default settings.

FRAP movies were acquired in confocal mode using a 40× plan-apochromat NA1.3 oil immersion objective at 13.3× zoom, corresponding to a 105-nm *XY* pixel size. JFX549 was excited with a 561-nm diode laser set at 0.25% with a master gain of 850 V, and the detector range was set at 566–635 nm. A 3D FRAP approach (Lemcke et al. 2016) was used to track the mobile MeCP2-enriched chromocenters, which would have otherwise moved out of focus from a single *Z*-plane during the long acquisition time. A circular bleach spot (*r* = 1 μm) was chosen to contain a nonsaturated chromocenter in its entirety. After acquiring five frames to measure baseline fluorescence, bleaching was obtained with two iterations of the 561-nm laser at maximal intensity. Bleaching was intentionally partial in order to track the target chromocenter any time after the bleaching step. Fluorescence recovery was recorded every ∼6.15 sec for ∼10 min, acquiring 13 *Z*-planes per frame (330-nm *Z* interval). We selected cells with similar MeCP2 expression levels in WT and G118E neurons so we could use the same illumination settings for both while avoiding signal saturation in WT cells. We analyzed data from 23 neurons dissected from five *Mecp2-Halo*^*WT*^^/y^ pups, 23 neurons dissected from 10 *Mecp2-Halo*^*G118E/y*^ pups, nine wild-type neurons infected with the Halo-NLS lentiviral vector dissected from two pups, and seven wild-type neurons infected with the H2B-Halo lentiviral vector from a single dissected pup.

Fast-SMT was performed on a custom-built Nikon microscope equipped with a 100×/NA1.49 oil immersion TIRF objective, an Andor iXon ultra EM-CCD camera, a perfect focus system, a motorized TIRF illuminator to achieve highly inclined and laminated optical sheet illumination (Tokunaga et al. 2008) an incubation chamber at 37°C with 5% CO_2_, and a heated objective (details described in Hansen et al. 2017). JFX549 was excited with a 561-nm laser (1 W; Genesis Coherent) to locate and focus cells, and PA-JF646 was photoactivated with a 405-nm laser (140 mW; OBIS, Coherent) and excited with a 639-nm laser (1 W; Genesis Coherent) to track single molecules. Fluorescence emission light was filtered using a Semrock 676/37-nm bandpass filter. The microscope, cameras, and hardware were controlled through NIS-Elements 4.60 software (Nikon). A region of interest (ROI) of 16 μm × 16 μm (100 pixels × 100 pixels), including the whole nucleus, was selected, and excitation and activation lasers were pulsed. Each frame consisted of a 7-msec camera exposure time followed by an ∼447-μsec camera “dead” time. In the first and last 20 frames, the 561-nm laser was pulsed for 7 msec to generate nuclear masks (see “Image Analysis”), taking advantage of JFX549 bleed-through in the 646 channel, while in the central 5000 frames the 633-nm excitation laser and the 405-nm activation laser were pulsed for 2 msec at the beginning of the camera exposure time and during the camera “dead” time, respectively. The photoactivation laser power was optimized to keep an average molecule density of approximately one localization per frame. Cells with spontaneous PA-JF646 activation were bleached with a short pulse (∼5 sec) of the 633-nm laser prior to acquisition. We recorded movies from a minimum of 20 cells per dissected animal from nine *Mecp2-Halo*^*WT*^^/y^ animals (206 cells; 279,505 total trajectories), nine *Mecp2-Halo*^*G118E/y*^ animals (201 cells; 231,950 total trajectories), one wild-type animal infected with the H2B-Halo lentiviral vector (38 cells; ∼35,000 total trajectories), and two wild-type animals infected with the Halo-NLS lentiviral vector (39 cells; ∼22,400 total trajectories).

Our live-cell imaging approach could not discriminate between different neuronal subtypes in the primary cultures, so our measurements were blind to the neuronal subtype and the maturation level of the cultured neurons, both of which might have impacted MeCP2 dynamics. However, we note that fast-SMT measurements probed >200 cells dissected from nine animals and a wide protein concentration range; thus, it is unlikely that they were biased by neuronal subtype and maturation state.

Raw images and movies are available in the Zenodo data repository (Zenodo, https://about.zenodo.org) under the following accession IDs: LSM900 Airyscan2 images (doi:10.5281/zenodo.7011051), 3D FRAP movies (doi:10.5281/zenodo.7017487), *Mecp2-Halo*^*WT*^^/y^ fast SPT (doi:10.5281/zenodo.7023479), *Mecp2-Halo*^*G118E*^^/y^ fast SPT (doi:10.5281/zenodo.7033279), H2B-Halo fast SPT (doi:10.5281/zenodo.7038507), and Halo-NLS fast SPT (doi:10.5281/zenodo.7048108).

### Image analysis

LSM 900 images were processed with Airyscan in 2D (H2B-Halo) or 3D (MeCP2-Halo) using Zen 3.1 blue edition software. For display purposes, minimum and maximum intensity values corresponding to the minimum/maximum values of the image with the broader intensity range were set equal for all images in each channel independently. Sixteen-bit images of either single planes or maximum intensity projections of 3D *Z*-stacks were converted to RGB and saved as .TIFF files.

To analyze 3D FRAP movies of WT and G118E MeCP2-Halo neurons, we first generated maximum intensity projections of *Z*-stacks. *Z*-projections were further projected in time, and an ROI enclosing the targeted chromocenter (including its movements in *Z* and time) was manually drawn (Supplemental Fig. S7B,C). This combined ROI was used to measure area and *Z*-projected (sum) mean fluorescence intensity of the target chromocenter at each time frame, which was used for fluorescence recovery calculations. We reasoned that this approach would give reasonable estimations of the fluorescence recovery of the moving MeCP2-Halo foci in *Z* and time. We used the same maximum *Z*/*t* projection approach to generate a mask comprising the whole nucleus and to select a dark region to measure background fluorescence. Nuclear masks were used to measure area and *Z*-projected (sum) mean nuclear fluorescence intensities of the whole nucleus at each time frame, which was used for photobleaching correction. Background regions were used to measure *Z*-projected (sum) mean background fluorescence intensities, which were used for background subtraction. For each cell and time frame, we subtracted the background fluorescence from both the chromocenter and the nuclear fluorescence. We then internally normalized the chromocenter and nuclear fluorescence intensities to the mean fluorescence intensity of the first five frames before bleaching. We finally corrected each cell for acquisition photobleaching by dividing the normalized chromocenter fluorescence intensities by the normalized nuclear fluorescence intensities frame by frame. Corrected FRAP curves from each single cell were averaged to generate a mean FRAP recovery, which was used to calculate the bleach depth (minimal intensity fluorescence [*I*_min_]), the maximal recovery (maximal intensity fluorescence [*I*_max_]), and the time to half-recovery (*t*_1/2_) as *t*_1/2_ = *I*_min_ + (*I*_max_ − *I*_min_) × 1/2.

We note that because our frame rate (∼6 sec) was long compared with the time scale of molecular diffusion, it was not possible to accurately estimate the bound and free fractions from our FRAP curves. Also, we refrained from kinetic modeling of our FRAP curves to estimate MeCP2 residence times due to the complex nature of chromocenters: Heterochromatic foci are dense and heavily methylated; thus, the apparent fluorescence recovery was likely a combination of long binding events and MeCP2 molecules that repeatedly unbound and rebound to chromatin before leaving the bleached chromocenter. We finally note that ∼30% of the acquired cells were not included in the analysis due to excessive nuclear movement, which caused chromocenters to either contact each other or move out of the focal plane during acquisition. The concentration of MeCP2-Halo at chromocenters and their appreciable movement also prevented FRAP measurements of MeCP2-Halo outside chromocenters. Therefore, drift correction and tracking of a bleached spot in the nucleoplasm in 3D would have been challenging and most likely inaccurate.

Fast-SMT movies were first processed with Fiji (Schindelin et al. 2012) with a custom macro to generate nuclear masks intersecting maximum *Z* projections of the first and last 20 frames acquired with 561-nm illumination. Masked movies were analyzed with the Quot package (https://github.com/alecheckert/quot) to localize single-molecule spots and connect them over time to generate trajectories with the following settings: [filter] start = 20, end = 5019, method = “identity,” chunk_size = 100; [detect] method = “llr,” *k* = 1.3, *w* = 15, *t* = 18.0; [localize] method = “ls_int_gaussian,” window_size = 9, σ = 1.0, ridge = 0.001, max_iter = 10, damp = 0.3; and [track] method = “euclidean,” pixel_ size_µm = 0.160, frame_interval = 0.00748, search_radius = 1, max_blinks = 0, min_I0 = 0.0, scale = 7.0. Movies with more than three average detections per frame were not included in downstream analyses.

Selected Quot output files were fed to the Spot-On package (https://spoton.berkeley.edu; Hansen et al. 2018) to obtain the distribution of single molecules’ jump lengths (i.e., the distance travelled by each molecule in a one-frame time interval).

Quot trajectories were then analyzed with the SASPT package (https://github.com/alecheckert/saspt) to infer the diffusion coefficient distribution of the experimental trajectories grouped by either animal or genotype (wild type or G118E) using the SASPT StateArrayDaset API with the following settings: pixel_size_µm = 0.16, frame_interval = 0.00748, focal_depth = 0.7, sample_size = 1000000, likelihood_type = “rbme,” and other default parameters. SASPT uses state arrays, a kind of variational Bayesian framework, to calculate the likelihood of diffusion coefficients for each trajectory, accounting for localization error and defocalization (for a detailed discussion, see Heckert et al. 2022).

The fraction of Halo-tagged proteins engaged in chromatin interactions (fraction bound) was calculated as the percent of molecules with a diffusion coefficient of <0.1 μm^2^/sec.

Scripts used for SMT and FRAP image analysis are available on request.

### Behavioral assays

All data acquisition and analyses were carried out by an individual blinded to the genotype and treatment. All behavioral studies were performed during the light period. Mice were habituated to the test room for 30 min before each test. At least 1 d was given between assays for the mice to recover. All tests were performed as previously described (Chao et al. 2010; Zhou et al. 2022) with few modifications. A detailed description is provided in the Supplemental Material.

#### LTP test

Induction and recording of hippocampal synaptic plasticity in vivo were conducted as previously published with a few modifications (Tang and Dani 2009; Hao et al. 2015, 2021). Mice were secured on a stereotaxic frame (David Kopf) and anesthetized with 1%–2% isoflurane. Under aseptic conditions, the recording electrode (Teflon-coated tungsten wire, bare diameter 50 µm; A-M Systems) was surgically aimed at the dentate gyrus (1.8–2.0 mm posterior and 1.4–1.6 mm lateral of bregma, 2.1–2.2 mm below the skull), while a concentric stimulating electrode (same sort of tungsten wire as for recordings) was implanted ipsilaterally in the medial perforant path (0.2 mm posterior and 2.8–3.0 mm lateral of λ, 1.0–1.3 mm below the dura). Evoked potentials of the performant path recorded in the dentate gyrus were used to guide the final positions of both electrodes. A cortical silver ball placed contralaterally served as ground. Dental cement was used to anchor the electrodes and the connecting device for chronic recordings. After at least 2 wk of recovery from surgical implantation, mice were transported and habituated to the recording system during each of the 4 d prior to starting the LTP test. Signals were picked up with a preamplifier (Pinnacle), amplified (100×), filtered (bandpass, 0.1–5 kHz), digitized at 10 kHz, and stored on disk for offline analysis (pClamp10 and 1440A; Molecular Devices). The time course of LTP tests was across 7 d (day −1 to day 5). Test responses elicited by monophasic pulses (0.1-msec duration) were recorded for 20-min periods on consecutive days at an intensity that evoked 40%–50% of the maximal population spike. Following 2 d of stable baseline, a tetanus (θ burst stimulation [TBS]) was delivered to the perforant path for LTP induction. Pulse width was doubled during tetani, which consisted of six series of six trains of six stimuli at 400 Hz with 200 msec between trains and 20 sec between series. Responses were measured for 60 min after tetanus and again for 20 min at 24, 48, and 120 h after tetanus. Since the latency of the population spike usually decreased following LTP induction, it was impractical to compare the initial slope of the fEPSP (field excitatory postsynaptic potential) before and after LTP induction in awake animals (Jones et al. 2001; Malleret et al. 2001). Accordingly, we quantified the amplitude of the population spikes (Tang and Dani 2009; Hao et al. 2015, 2021). Data were averaged every 5 min and normalized to the baseline measured over the 10 min before tetanic stimulation and are presented as mean ± standard error of mean. Two-way repeated measures ANOVA (between groups) or one-way ANOVA (within group) was used for data analysis.

#### Video*-*EEG and data analysis

Mice were anesthetized with 2% isoflurane and mounted in a stereotaxic frame (David Kopf Instruments). Under aseptic conditions, each mouse was implanted with cortical recording electrodes (Teflon-coated silver wire, bare diameter 125 µm; A-M Systems) aimed at the subdural space of the somatosensory cortex (0.8 mm posterior and 1.8 mm lateral to bregma) and the frontal cortex (1.8 mm anterior and 1.5 mm lateral to bregma), respectively. The reference electrode was then positioned in the occipital region of the skull. The third recording electrode (constructed with Teflon-coated tungsten wire, bare diameter 50 µm; A-M systems) was stereotaxically implanted in the CA1 of the hippocampus (1.9 mm posterior , 1.0 mm lateral, and 1.3 mm ventral to bregma) (Paxinos and Franklin 2001) with reference to the ipsilateral corpus callosum for local field potential (LFP) recordings. All electrode wires were attached to a miniature connector (Harwin Connector). After 2 wk of postsurgical recovery, EEG and LFP signals (filtered between 0.5 Hz and 1 kHz; sampled at 2 kHz) were amplified (100×) by a 1700 Differential AC amplifier (A-M Systems) and recorded by a DigiData 1440A and pCLAMP 10 data acquisition system (Molecular Devices, LLC) for offline data analysis. The synchronized mouse behavior was recorded with an ANY-maze tracking system (Stoelting Co.). The video-EEG/LFP recordings were conducted for 2 h per day over 3 d (Wang et al. 2022).

Abnormal synchronous discharges were manually identified when the sharp positive deflections exceeded twice the baseline and lasted 25–100 msec (Roberson et al. 2011). The numbers of abnormal spikes over the recording period were counted using Clampfit 10 software (Molecular Devices, LLC). The spike numbers across sessions were averaged per animal and statistically compared between genotypes.

#### Statistical analysis

Statistical significance was analyzed using GraphPad Prism. The number of animals (*n*) and the specific statistical tests for each experiment are indicated in the figure legends. Sample size for behavioral studies was determined based on previous reports using transgenic mice with the same background. Mice were randomly assigned using Excel software to generate a table of random numbers, and the experimenter was always blinded to the treatment. For behavioral assays, all population values appeared to be normally distributed. Equal variances were never assumed, and the Geisser–Greenhouse correction for sphericity was always applied when using ANOVA.

### Data and material availability

All data needed to evaluate the conclusions in this study are present in here and/or the in the Supplemental Material. Raw imaging data are available in the Zenodo data repository (see the Materials and Methods). CUT&RUN raw sequencing reads and processed data were deposited to GEO (GSE243009).

## Supplementary Material

Supplement 1

## References

[GAD350733ZHOC1] Amir RE, van den Veyver IB, Wan M, Tran CQ, Francke U, Zoghbi HY. 1999. Rett syndrome is caused by mutations in X-linked MECP2, encoding methyl- CpG-binding protein 2. Nat Genet 23: 185–188. 10.1038/13810 10508514

[GAD350733ZHOC2] Bajikar SS, Anderson AG, Zhou J, Durham MA, Trostle AJ, Wan Y-W, Liu Z, Zoghbi HY. 2023. MeCP2 regulates Gdf11, a dosage-sensitive gene critical for neurological function. Elife 12: e83806. 10.7554/eLife.83806 36848184 PMC9977283

[GAD350733ZHOC3] Baker SA, Chen L, Wilkins AD, Yu P, Lichtarge O, Zoghbi HY. 2013. A AT-hook domain in MeCP2 determines the clinical course of Rett syndrome and related disorders. Cell 152: 984–996. 10.1016/j.cell.2013.01.038 23452848 PMC3641682

[GAD350733ZHOC4] Brown K, Selfridge J, Lagger S, Connelly J, de Sousa D, Kerr A, Webb S, Guy J, Merusi C, Koerner MV, 2016. The molecular basis of variable phenotypic severity among common missense mutations causing Rett syndrome. Hum Mol Genet 25: 558–570. 10.1093/hmg/ddv496 26647311 PMC4731022

[GAD350733ZHOC5] Chahrour M, Zoghbi HY. 2007. The story of Rett syndrome: from clinic to neurobiology. Neuron 56: 422–437. 10.1016/j.neuron.2007.10.001 17988628

[GAD350733ZHOC6] Chao HT, Chen H, Samaco RC, Xue M, Chahrour M, Yoo J, Neul JL, Gong S, Lu HC, Heintz N, 2010. Dysfunction in GABA signalling mediates autism-like stereotypies and Rett syndrome phenotypes. Nature 468: 263–269. 10.1038/nature09582 21068835 PMC3057962

[GAD350733ZHOC7] Chen L, Chen K, Lavery LA, Baker SA, Shaw CA, Li W, Zoghbi HY. 2015. MeCP2 binds to non-CG methylated DNA as neurons mature, influencing transcription and the timing of onset for Rett syndrome. Proc Natl Acad Sci 112: 5509–5514. 10.1073/pnas.1505909112 25870282 PMC4418849

[GAD350733ZHOC8] Chen Y, Cattoglio C, Dailey GM, Zhu Q, Tjian R, Darzacq X. 2022. Mechanisms governing target search and binding dynamics of hypoxia-inducible factors. Elife 11: e75064. 10.7554/eLife.75064 36322456 PMC9681212

[GAD350733ZHOC9] Chong S, Dugast-Darzacq C, Liu Z, Dong P, Dailey GM, Cattoglio C, Heckert A, Banala S, Lavis L, Darzacq X, 2018. Imaging dynamic and selective low-complexity domain interactions that control gene transcription. Science 361: eaar2555. 10.1126/science.aar2555 29930090 PMC6961784

[GAD350733ZHOC10] Crutcher E, Pal R, Naini F, Zhang P, Laugsch M, Kim J, Bajic A, Schaaf CP. 2019. mTOR and autophagy pathways are dysregulated in murine and human models of Schaaf-Yang syndrome. Sci Rep 9: 15935. 10.1038/s41598-019-52287-2 31685878 PMC6828689

[GAD350733ZHOC11] Gandaglia A, Brivio E, Carli S, Palmieri M, Bedogni F, Stefanelli G, Bergo A, Leva B, Cattaneo C, Pizzamiglio L, 2019. A novel *Mecp2*^*Y120D*^ knock-in model displays similar behavioral traits but distinct molecular features compared to the *Mecp2*-null mouse implying precision medicine for the treatment of Rett syndrome. Mol Neurobiol 56: 4838–4854. 10.1007/s12035-018-1412-2 30402709

[GAD350733ZHOC12] Georgel PT, Horowitz-Scherer RA, Adkins N, Woodcock CL, Wade PA, Hansen JC. 2003. Chromatin compaction by human MeCP2: assembly of novel secondary chromatin structures in the absence of DNA methylation. J Biol Chem 278: 32181–32188. 10.1074/jbc.M305308200 12788925

[GAD350733ZHOC13] Glaze DG, Percy AK, Skinner S, Motil KJ, Neul JL, Barrish JO, Lane JB, Geerts SP, Annese F, Graham J, 2010. Epilepsy and the natural history of Rett syndrome. Neurology 74: 909–912. 10.1212/WNL.0b013e3181d6b852 20231667 PMC2836870

[GAD350733ZHOC14] Goffin D, Allen M, Zhang L, Amorim M, Wang ITJ, Reyes ARS, Mercado-Berton A, Ong C, Cohen S, Hu L, 2012. Rett syndrome mutation MeCP2 T158A disrupts DNA binding, protein stability and ERP responses. Nat Neurosci 15: 274–283. 10.1038/nn.2997 PMC3267879 22119903

[GAD350733ZHOC015] Grimm JB, Xie L, Casler JC, Patel R, Tkachuk AN, Falco N, Choi H, Lippincott-Schwartz J, Brown TA, Glick BS, 2021. A general method to improve fluorophores using deuterated auxochromes. JACS Au 1: 690–696. 10.1021/jacsau.1c00006 34056637 PMC8154212

[GAD350733ZHOC15] Guy J, Hendrich B, Holmes M, Martin JE, Bird A. 2001. A mouse Mecp2-null mutation causes neurological symptoms that mimic rett syndrome. Nat Genet 27: 322–326. 10.1038/85899 11242117

[GAD350733ZHOC16] Guy J, Gan J, Selfridge J, Cobb S, Bird A. 2007. Reversal of neurological defects in a mouse model of Rett syndrome. Science 315: 1143–1147. 10.1126/science.1138389 17289941 PMC7610836

[GAD350733ZHOC17] Hansen AS, Pustova I, Cattoglio C, Tjian R, Darzacq X. 2017. CTCF and cohesin regulate chromatin loop stability with distinct dynamics. Elife 6: e25776. 10.7554/eLife.25776 28467304 PMC5446243

[GAD350733ZHOC18] Hansen AS, Woringer M, Grimm JB, Lavis LD, Tjian R, Darzacq X. 2018. Robust model-based analysis of single-particle tracking experiments with Spot-On. Elife 7: e33125. 10.7554/eLife.33125 29300163 PMC5809147

[GAD350733ZHOC19] Hao S, Tang B, Wu Z, Ure K, Sun Y, Tao H, Gao Y, Patel AJ, Curry DJ, Samaco RC, 2015. Forniceal deep brain stimulation rescues hippocampal memory in Rett syndrome mice. Nature 526: 430–434. 10.1038/nature15694 26469053 PMC4828032

[GAD350733ZHOC20] Hao S, Wang Q, Tang B, Wu Z, Yang T, Tang J. 2021. CDKL5 deficiency augments inhibitory input into the dentate gyrus that can be reversed by deep brain stimulation. J Neurosci 41: 9031–9046. 10.1523/JNEUROSCI.1010-21.2021 34544833 PMC8549531

[GAD350733ZHOC21] Heckert A, Dahal L, Tijan R, Darzacq X. 2022. Recovering mixtures of fast-diffusing states from short single-particle trajectories. Elife 11: e70169. 10.7554/eLife.70169 36066004 PMC9451534

[GAD350733ZHOC22] Heckman LD, Chahrour MH, Zoghbi HY. 2014. Rett-causing mutations reveal two domains critical for MeCP2 function and for toxicity in MECP2 duplication syndrome mice. Elife 3: 1–17. 10.7554/eLife.02676 PMC4102243 24970834

[GAD350733ZHOC23] Ho KL, McNae IW, Schmiedeberg L, Klose RJ, Bird AP, Walkinshaw MD. 2008. MeCP2 binding to DNA depends upon hydration at methyl-CpG. Mol Cell 29: 525–531. 10.1016/j.molcel.2007.12.028 18313390

[GAD350733ZHOC24] Ito-Ishida A, Yamalanchili HK, Shao Y, Baker SA, Heckman LD, Lavery LA, Kim JY, Lombardi LM, Sun Y, Liu Z, 2018. Genome-wide distribution of linker histone H1.0 is independent of MeCP2. Nat Neurosci 21: 794–798. 10.1038/s41593-018-0155-8 29802390 PMC6099063

[GAD350733ZHOC25] Ito-Ishida A, Baker SA, Sillitoe RV, Sun Y, Zhou J, Ono Y, Iwakiri J, Yuzaki M, Zoghbi HY. 2020. MeCP2 levels regulate the 3d structure of heterochromatic foci in mouse neurons. J Neurosci 40: 8746–8766. 10.1523/JNEUROSCI.1281-19.2020 33046553 PMC7643291

[GAD350733ZHOC26] Jentarra GM, Olfers SL, Rice SG, Srivastava N, Homanics GE, Blue M, Naidu SB, Narayanan V. 2010. Abnormalities of cell packing density and dendritic complexity in the MeCP2 A140V mouse model of Rett syndrome/X-linked mental retardation. BMC Neurosci 11: 1–15. 10.1186/1471-2202-11-19 PMC2836362 20163734

[GAD350733ZHOC27] Jiang X, Chen J, Bajić A, Zhang C, Song X, Carroll SL, Cai ZL, Tang M, Xue M, Cheng N, 2017. Quantitative real-time imaging of glutathione. Nat Commun 8: 1–13. 10.1038/ncomms16087 28703127 PMC5511354

[GAD350733ZHOC28] Johnson BS, Zhao YT, Fasolino M, Lamonica JM, Kim YJ, Georgakilas G, Wood KH, Bu D, Cui Y, Goffin D, 2017. Biotin tagging of MeCP2 in mice reveals contextual insights into the Rett syndrome transcriptome. Nat Med 23: 1203–1214. 10.1038/nm.4406 28920956 PMC5630512

[GAD350733ZHOC29] Jones MW, Errington ML, French PJ, Fine A, Bliss TVP, Garel S, Charnay P, Bozon B, Laroche S, Davis S. 2001. A requirement for the immediate early gene Zif268 in the expression of late LTP and long-term memories. Nat Neurosci 4: 289–296. 10.1038/85138 11224546

[GAD350733ZHOC30] Lamonica JM, Kwon DY, Goffin D, Fenik P, Johnson BS, Cui Y, Guo H, Veasey S, Zhou Z. 2017. Elevating expression of MeCP2 T158M rescues DNA binding and Rett syndrome-like phenotypes. J Clin Invest 127: 1889–1904. 10.1172/JCI90967 28394263 PMC5409785

[GAD350733ZHOC31] Laurvick CL, de Klerk N, Bower C, Christodoulou J, Ravine D, Ellaway C, Williamson S, Leonard H. 2006. Rett syndrome in Australia: a review of the epidemiology. J Pediatr 148: 347–352. 10.1016/j.jpeds.2005.10.037 16615965

[GAD350733ZHOC32] Lavery LA, Ure K, Wan YW, Luo C, Trostle AJ, Wang W, Jin H, Lopez J, Lucero J, Durham MA, 2020. Losing dnmt3a dependent methylation in inhibitory neurons impairs neural function by a mechanism impacting rett syndrome. Elife 9: e52981. 10.7554/eLife.52981 32159514 PMC7065908

[GAD350733ZHOC33] Lemcke H, Peukert J, Voronina N, Skorska A, Steinhoff G, David R. 2016. Applying 3D-FRAP microscopy to analyse gap junction-dependent shuttling of small antisense RNAs between cardiomyocytes. J Mol Cell Cardiol 98: 117–127. 10.1016/j.yjmcc.2016.07.008 27480520

[GAD350733ZHOC34] Liu Z, Tjian R. 2018. Visualizing transcription factor dynamics in living cells. J Cell Biol 217: 1181–1191. 10.1083/jcb.201710038 29378780 PMC5881510

[GAD350733ZHOC35] Liu YL, Horning AM, Lieberman B, Kim M, Lin CK, Hung CN, Chou CW, Wang CM, Lin CL, Kirma NB, 2019. Spatial EGFR dynamics and metastatic phenotypes modulated by upregulated EphB2 and Src pathways in advanced prostate cancer. Cancers (Basel) 11: 1910. 10.3390/cancers11121910 31805710 PMC6966510

[GAD350733ZHOC36] Malenka RC, Bear MF. 2004. LTP and LTD: an embarrassment of riches. Neuron 44: 5–21. 10.1016/j.neuron.2004.09.012 15450156

[GAD350733ZHOC37] Malleret G, Haditsch U, Genoux D, Jones MW, Bliss TVP, Vanhoose AM, Weitlauf C, Kandel ER, Winder DG, Mansuy IM. 2001. Inducible and reversible enhancement of learning, memory, and long-term potentiation by genetic inhibition of calcineurin. Cell 104: 675–686. 10.1016/S0092-8674(01)00264-1 11257222

[GAD350733ZHOC38] Meehan R, Lewis JD, Bird AP. 1992. Characterization of MECP2, a vertebrate DNA binding protein with affinity for methylated DNA. Nucleic Acids Res 20: 5085–5092. 10.1093/nar/20.19.5085 1408825 PMC334288

[GAD350733ZHOC39] Moser EI, Krobert KA, Moser MB, Morris RGM. 1998. Impaired spatial learning after saturation of long-term potentiation. Science 281: 2038–2042. 10.1126/science.281.5385.2038 9748165

[GAD350733ZHOC40] Nan X, Meehan RR, Bird A. 1993. Dissection of the methyl-CpG binding domain from the chromosomal protein MeCP2. Nucleic Acids Res 21: 4886–4892. 10.1093/nar/21.21.4886 8177735 PMC311401

[GAD350733ZHOC41] Nan X, Tate P, Li E, Bird A. 1996. DNA methylation specifies chromosomal localization of MeCP2. Mol Cell Biol 16: 414–421. 10.1128/MCB.16.1.414 8524323 PMC231017

[GAD350733ZHOC42] Neul JL, Benke TA, Marsh ED, Skinner SA, Merritt J, Lieberman DN, Standridge S, Feyma T, Heydemann P, Peters S, 2019. The array of clinical phenotypes of males with mutations in *methyl-CpG binding protein 2*. Am J Med Genet B Neuropsychiatr Genet 180: 55–67. 10.1002/ajmg.b.32707 30536762 PMC6488031

[GAD350733ZHOC43] Nikitina T, Shi X, Ghosh RP, Horowitz-Scherer RA, Hansen JC, Woodcock CL. 2007. Multiple modes of interaction between the methylated DNA binding protein MeCP2 and chromatin. Mol Cell Biol 27: 864–877. 10.1128/MCB.01593-06 17101771 PMC1800686

[GAD350733ZHOC044] Pan H, Mostoslavsky G, Eruslanov E, Kotton DN, Kramnik I. 2008. Dual-promoter lentiviral system allows inducible expression of noxious proteins in macrophages. J Immunol Methods 329: 31–44. 10.1016/j.jim.2007.09.009 17967462 PMC2244810

[GAD350733ZHOC44] Parolia A, Cieslik M, Chu SC, Xiao L, Ouchi T, Zhang Y, Wang X, Vats P, Cao X, Pitchiaya S, 2019. Distinct structural classes of activating FOXA1 alterations in advanced prostate cancer. Nature 571: 413–418. 10.1038/s41586-019-1347-4 31243372 PMC6661908

[GAD350733ZHOC45] Paxinos G, Franklin KBJ. 2001. Paxinos and Franklin's the mouse brain in stereotaxic coordinates, 2nd ed. Academic Press, San Diego.

[GAD350733ZHOC46] Piccolo FM, Liu Z, Dong P, Hsu CL, Stoyanova EI, Rao A, Tjian R, Heintz N. 2019. MeCP2 nuclear dynamics in live neurons results from low and high affinity chromatin interactions. Elife 8: e51449. 10.7554/eLife.51449 31868585 PMC6957317

[GAD350733ZHOC47] Roberson ED, Halabisky B, Yoo JW, Yao J, Chin J, Yan F, Wu T, Hamto P, Devidze N, Yu GQ, 2011. Amyloid-β/Fyn-induced synaptic, network, and cognitive impairments depend on tau levels in multiple mouse models of Alzheimer's disease. J Neurosci 31: 700–711. 10.1523/jneurosci.4152-10.2011 21228179 PMC3325794

[GAD350733ZHOC48] Rousseaux MWC, Tschumperlin T, Lu HC, Lackey EP, Bondar VV, Wan YW, Tan Q, Adamski CJ, Friedrich J, Twaroski K, 2018a. ATXN1–CIC complex is the primary driver of cerebellar pathology in spinocerebellar ataxia type 1 through a gain-of-function mechanism. Neuron 97: 1235–1243.e5. 10.1016/j.neuron.2018.02.013 29526553 PMC6422678

[GAD350733ZHOC49] Rousseaux MWC, Vázquez-Vélez GE, Al-Ramahi I, Jeong HH, Bajić A, Revelli JP, Ye H, Phan ET, Deger JM, Perez AM, 2018b. A druggable genome screen identifies modifiers of α-synuclein levels via a tiered cross-species validation approach. J Neurosci 38: 9286–9301. 10.1523/JNEUROSCI.0254-18.2018 30249792 PMC6199406

[GAD350733ZHOC50] Samaco RC, Mcgraw CM, Ward CS, Sun Y, Neul JL, Zoghbi HY. 2013. Female *Mecp2*^+/−^ mice display robust behavioral deficits on two different genetic backgrounds providing a framework for pre-clinical studies. Hum Mol Genet 22: 96–109. 10.1093/hmg/dds406 23026749 PMC3522402

[GAD350733ZHOC51] Schindelin J, Arganda-Carreras I, Frise E, Kaynig V, Longair M, Pietzsch T, Preibisch S, Rueden C, Saalfeld S, Schmid B, 2012. Fiji: an open-source platform for biological-image analysis. Nat Methods 9: 676–682. 10.1038/nmeth.2019 22743772 PMC3855844

[GAD350733ZHOC52] Skene PJ, Henikoff S. 2017. An efficient targeted nuclease strategy for high-resolution mapping of DNA binding sites. Elife 6: e21856. 10.7554/eLife.21856 28079019 PMC5310842

[GAD350733ZHOC53] Tang J, Dani JA. 2009. Dopamine enables in vivo synaptic plasticity associated with the addictive drug nicotine. Neuron 63: 673–682. 10.1016/j.neuron.2009.07.025 19755109 PMC2746116

[GAD350733ZHOC54] Tarquinio DC, Motil KJ, Hou W, Lee HS, Glaze DG, Skinner SA, Neul JL, Annese F, McNair L, Barrish JO, 2012. Growth failure and outcome in Rett syndrome: specific growth references. Neurology 79: 1653–1661. 10.1212/WNL.0b013e31826e9a70 23035069 PMC3468773

[GAD350733ZHOC55] Tokunaga M, Imamoto N, Sakata-Sogawa K. 2008. Highly inclined thin illumination enables clear single-molecule imaging in cells. Nat Methods 5: 159–161. 10.1038/nmeth1171 18176568

[GAD350733ZHOC56] Vázquez-Vélez GE, Gonzales KA, Revelli JP, Adamski CJ, Alavi Naini F, Bajić A, Craigen E, Richman R, Heman-Ackah SM, Wood MJA, 2020. Doublecortin-like kinase 1 regulates α-synuclein levels and toxicity. J Neurosci 40: 459–477. 10.1523/JNEUROSCI.1076-19.2019 31748376 PMC6948939

[GAD350733ZHOC57] Wang L, Hu M, Zuo MQ, Zhao J, Wu D, Huang L, Wen Y, Li Y, Chen P, Bao X, 2020. Rett syndrome-causing mutations compromise MeCP2-mediated liquid–liquid phase separation of chromatin. Cell Res 30: 393–407. 10.1038/s41422-020-0288-7 32111972 PMC7196128

[GAD350733ZHOC58] Wang Q, de Prisco N, Tang J, Gennarino VA. 2022. Protocol for recording epileptiform discharges of EEG and behavioral seizures in freely moving mice. STAR Protoc 3: 101245. 10.1016/j.xpro.2022.101245 35310070 PMC8927982

[GAD350733ZHOC59] Whitlock JR, Heynen AJ, Shuler MG, Bear MF. 2006. Learning induces long-term potentiation in the hippocampus. Science 313: 1093–1097. 10.1126/science.1128134 16931756

[GAD350733ZHOC60] Winnepenninckx B, Errijgers V, Hayez-Delatte F, Reyniers E, Kooy RF. 2002. Identification of a family with nonspecific mental retardation (MRX79) with the A140V mutation in the MECP2 gene: is there a need for routine screening? Hum Mutat 20: 249–252. 10.1002/humu.10130 12325019

[GAD350733ZHOC061] Wolterink-Donselaar IG, Meerding JM, Fernandes C. 2009. A method for gender determination in newborn dark pigmented mice. Lab Anim 38: 35–38. 10.1038/laban0109-35 19112448

[GAD350733ZHOC61] Zhang Y, Pak CH, Han Y, Ahlenius H, Zhang Z, Chanda S, Marro S, Patzke C, Acuna C, Covy J, 2013. Rapid single-step induction of functional neurons from human pluripotent stem cells. Neuron 78: 785–798. 10.1016/j.neuron.2013.05.029 23764284 PMC3751803

[GAD350733ZHOC62] Zhang H, Romero H, Schmidt A, Gagova K, Qin W, Bertulat B, Lehmkuhl A, Milden M, Eck M, Meckel T, 2022a. MeCP2-induced heterochromatin organization is driven by oligomerization-based liquid–liquid phase separation and restricted by DNA methylation. Nucleus 13: 1–34. 10.1080/19491034.2021.2024691 35156529 PMC8855868

[GAD350733ZHOC63] Zhang X, Cattoglio C, Zoltek M, Vetralla C, Mozumdar D, Schepartz A. 2022b. Dose-dependent nuclear delivery and transcriptional repression with a cell-penetrant MeCP2. ACS Cent Sci 17: 32. 10.1021/acscentsci.2c01226 PMC9951310 36844491

[GAD350733ZHOC64] Zhou J, Hamdan H, Yalamanchili HK, Pang K, Pohodich AE, Lopez J, Shao Y, Oses-Prieto JA, Li L, Kim W, 2022. Disruption of MeCP2–TCF20 complex underlies distinct neurodevelopmental disorders. Proc Natl Acad Sci 119: e2119078119. 10.1073/pnas.2119078119 35074918 PMC8794850

