# Supplementary material for: A novel pathogenic mutation of MeCP2 impairs chromatin association independent of protein levels
Source: Genes Dev. 2023 Oct 1;37(19-20):883–900. doi: 10.1101/gad.350733.123 (PMC10691473; doi:10.1101/gad.350733.123)
Supplement: Supplement 1 [file Supplemental_Data.docx]

**Supplementary Figures**


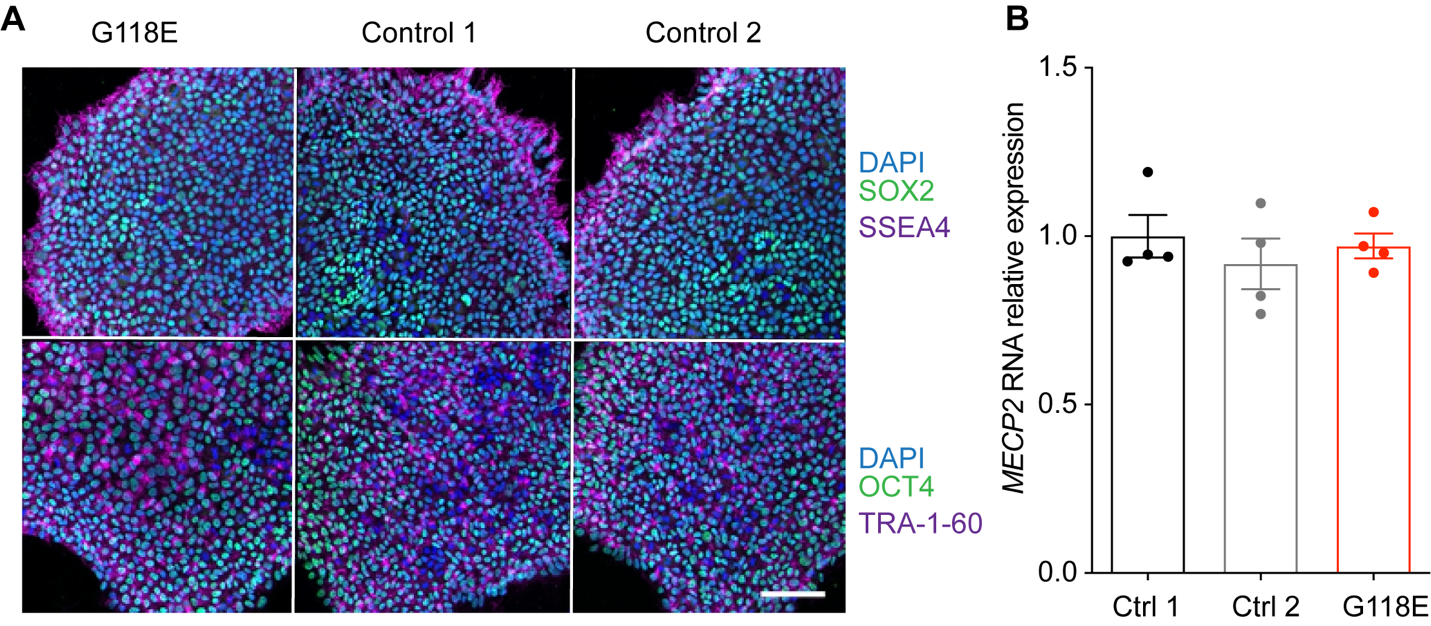


**Supplemental Fig. S1. Generation of patient fibroblast-derived iPSCs and isogenic controls. (A).** Immunostaining on induced pluripotent stem cells markers in the G118E line and two independent isogenic control lines. Top row: colonies stained with DAPI (blue) and against SOX2 (green) and SSEA4 (magenta). Bottom row: colonies stained with DAPI (blue) and against OCT4 (green) and TRA-1-60 (magenta). Scale bar: 100 µm. **(B).** Quantitative PCR on *MECP2* RNA in the G118E line and two independent isogenic control lines.

**
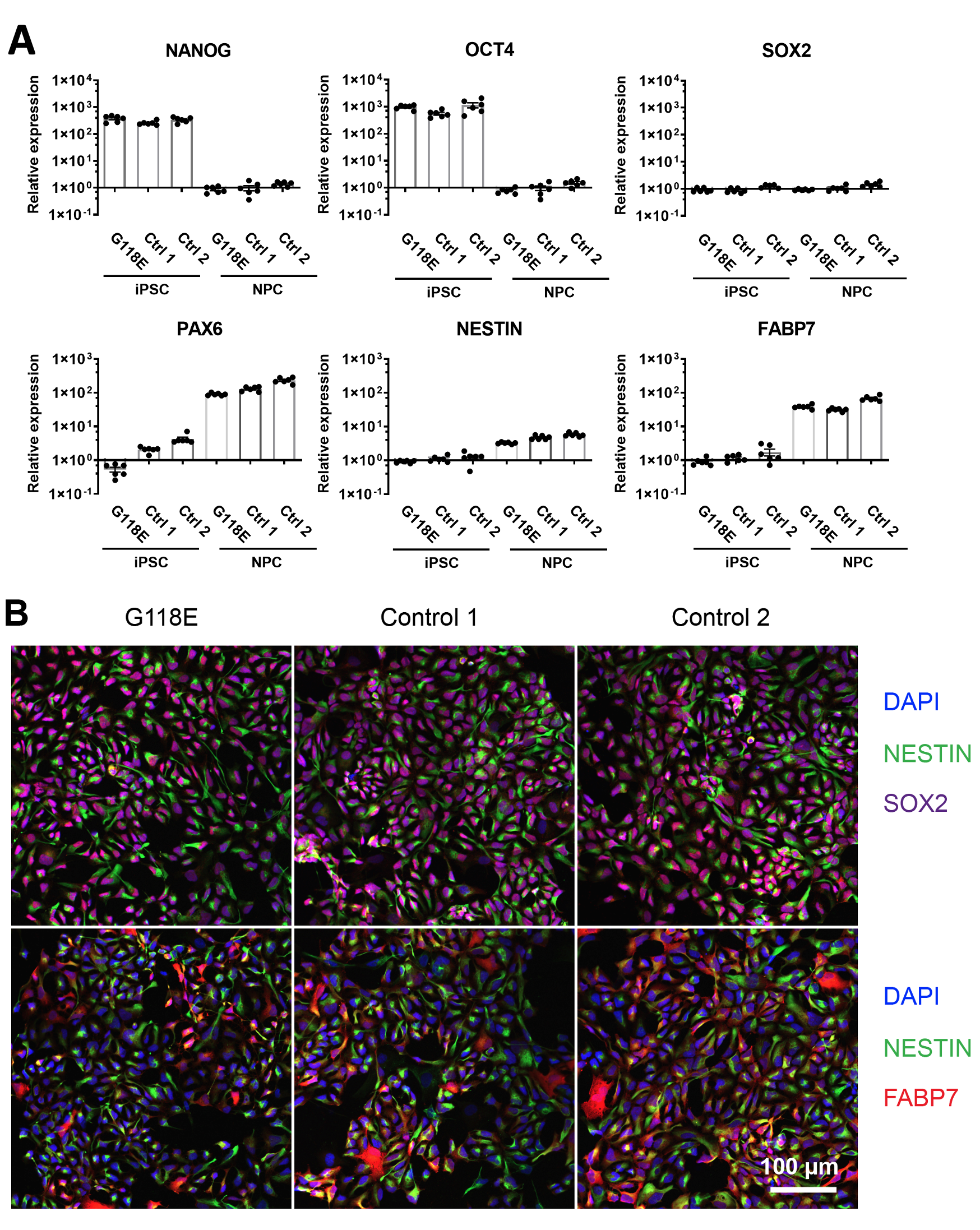
**

**Supplemental Fig. S2. Generation of neural progenitor cells from G118E iPSCs and isogenic controls. (A).** Quantitative PCR on induced pluripotent stem cells and neural progenitor cells markers in the G118E line and two independent isogenic control lines. **(B).** Immunostaining on induced pluripotent stem cells markers in two independent isogenic control lines and the G118E line. Top row: cells stained with DAPI (blue) and against NESTIN (green) and SOX2 (magenta). Bottom row: colonies stained with DAPI (blue) and against NESTIN (green) and FABP7 (red). Scale bar: 100 µm. Each culture represents a mix of NPCs from two independent inductions.

**
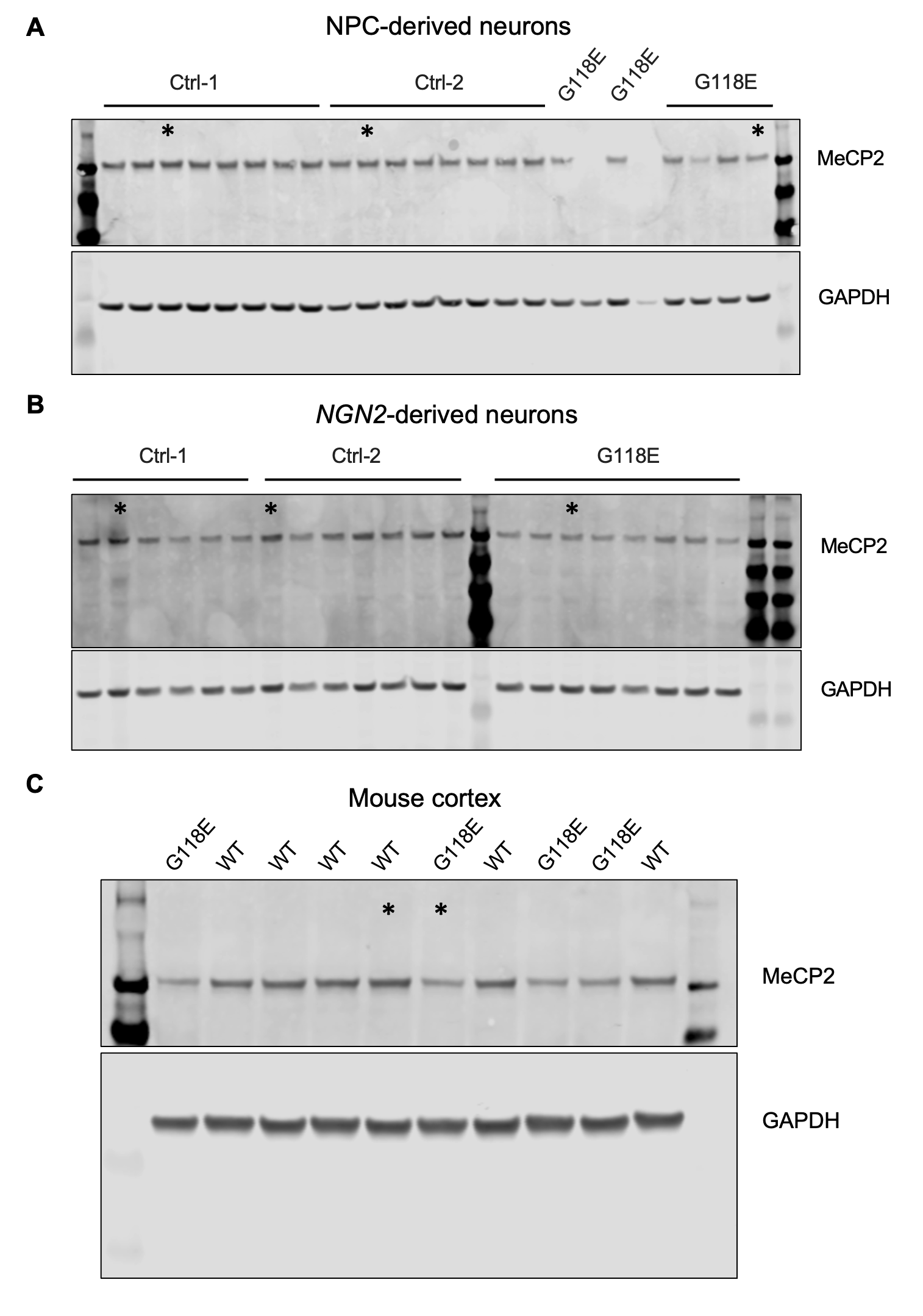
**

**Supplemental Fig. S3. Original western blots of MeCP2 protein in the patient fibroblast-derived neurons and mouse brain. (A)** Western blot of MeCP2 protein in neurons derived from neural progenitor cells (n=6-8). Asterisks denote the lanes displayed in **Fig. 1B**. **(B)** Western blot and quantification of MeCP2 protein in neurons derived directly by NGN2 overexpression (n=6-8). Asterisks denote the lanes displayed in **Fig. 1C**. **(C)** Western blot on MeCP2 protein in 8-week-old WT and *Mecp2^G118E/y^* mice in the cortex (n=4-6). Asterisks denote the lanes displayed in **Fig. 2B**.


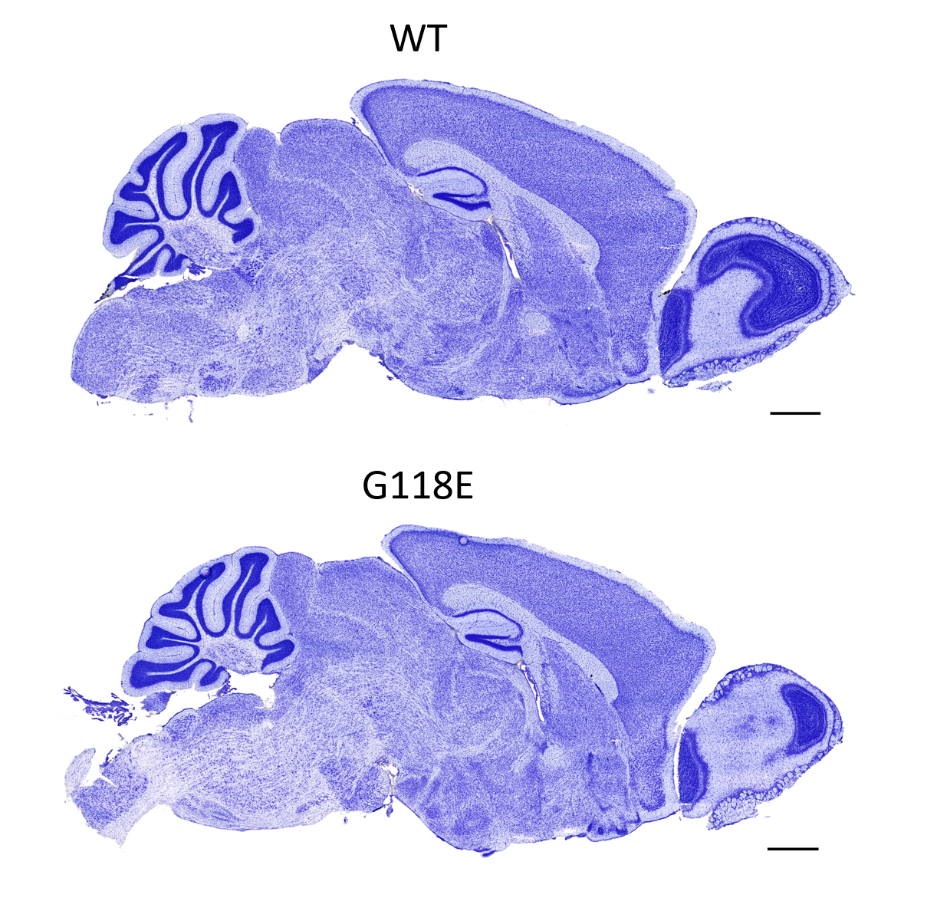
**Supplemental Fig. S4. *Mecp2^G118E/y^* mice show no gross changes in anatomy or structure in the brain.** Images of Cresyl violet stain of sagittal sections from wild-type or *Mecp2^G118E/y^* brains. Scale bar is 1 mm.

**
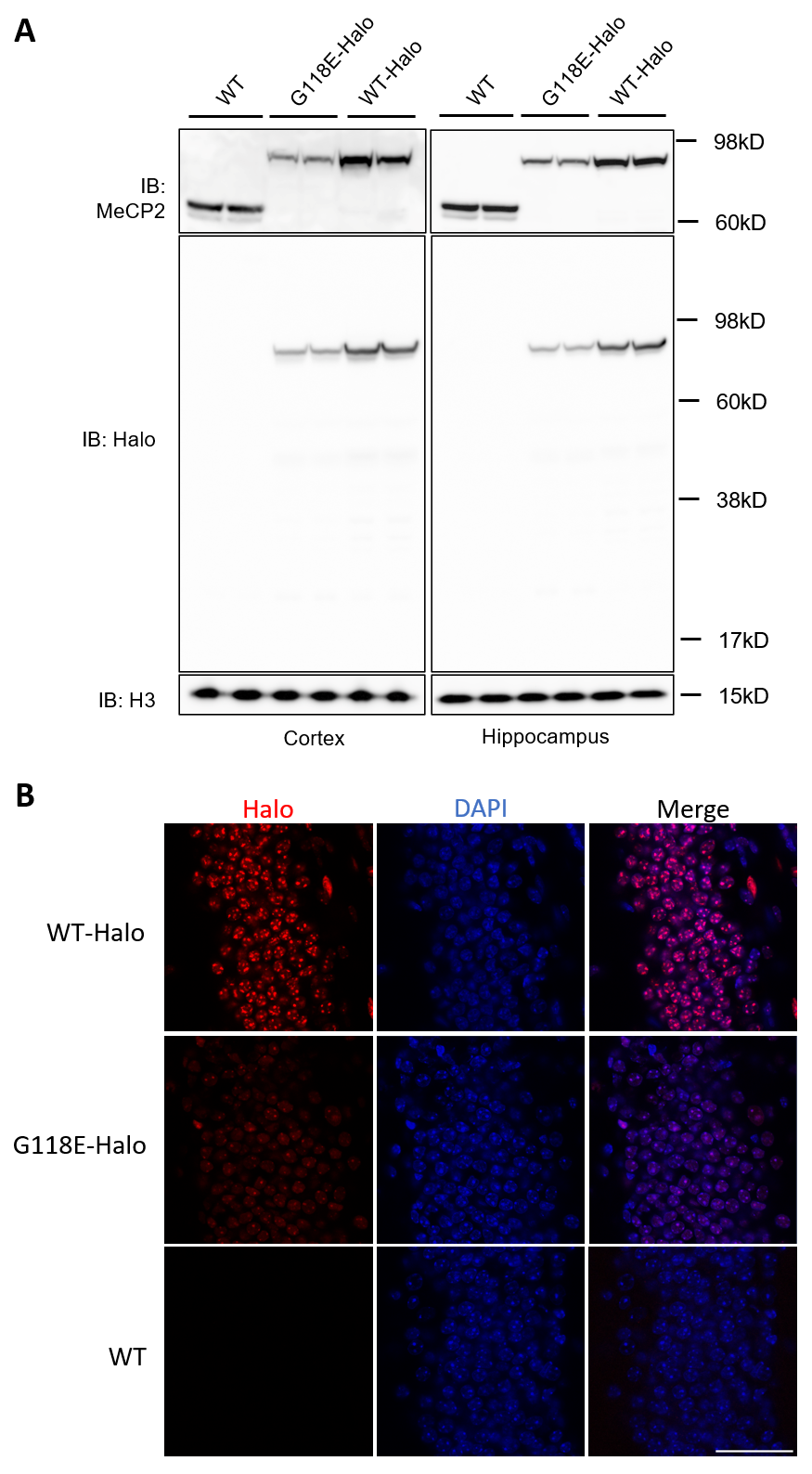
**

**Supplemental Fig. S5. Halo-tagged MeCP2 protein expresses in *Mecp2-Halo* and *Mecp2^G118E^-Halo* mice brain. (A)** Western blot detects Halo-tagged MeCP2 using anti-MeCP2 and anti-Halo antibodies in *Mecp2-Halo* and *Mecp2^G118E^-Halo* mice in the cortex (left) and hippocampus (right). **(B)** Immunofluorescence staining using HaloTag Ligand showed Halo-tagged MeCP2 binding to heterochromatin foci in *Mecp2-Halo* and *Mecp2^G118E^-Halo* mice in hippocampus. Scale bar: 50 μm.


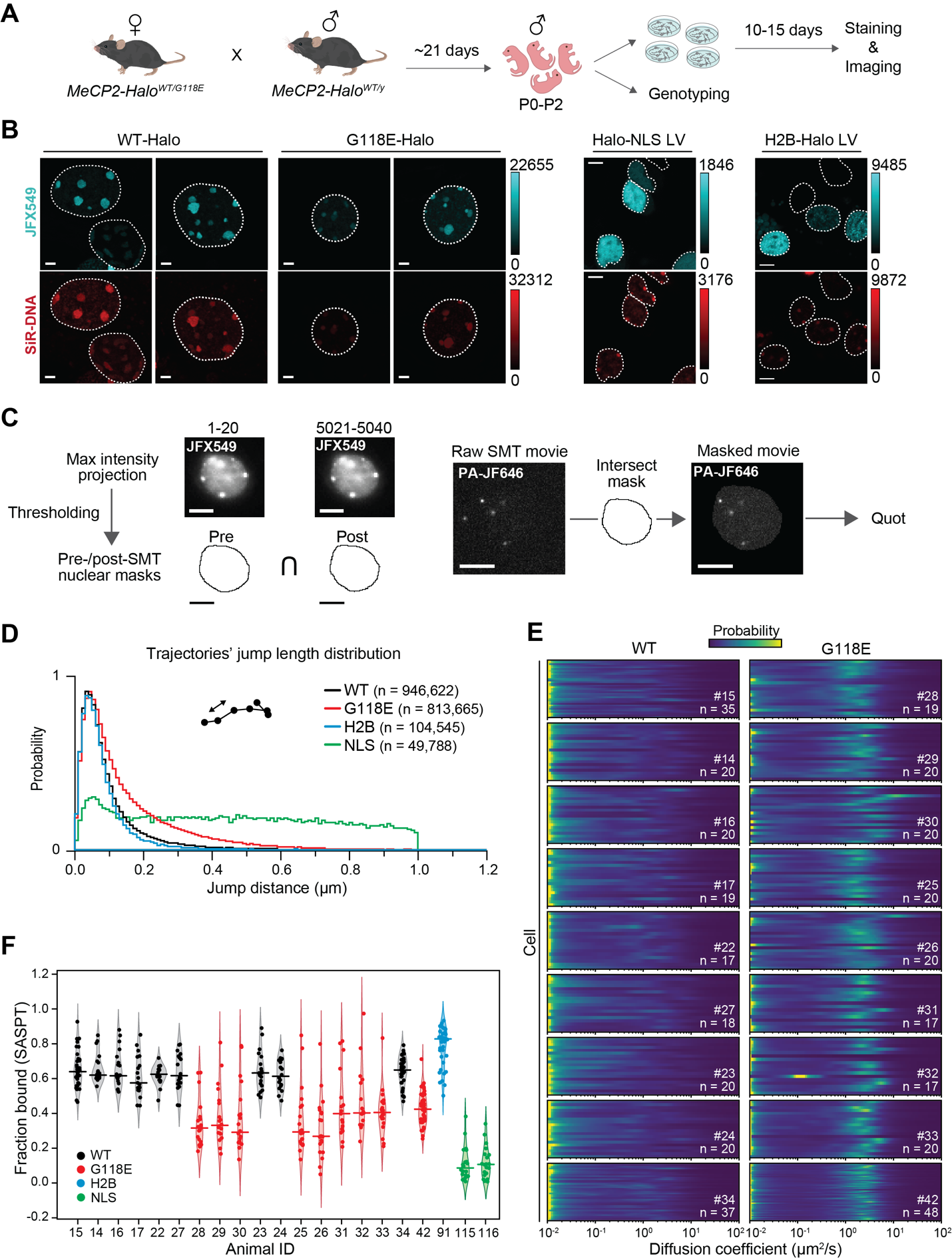


**Supplemental Fig. S6. Measuring MeCP2-Halo nuclear dynamics in primary cortical neurons. (A)** Mating scheme to obtain both *Mecp2-Halo* and *MeCP2^G118E^-Halo* male pups from the same litter. Litters were dissected between postnatal day 0 (P0) and P2 to isolate cortical neurons, and matured in culture for 12-15 days prior to staining and imaging. Pups gender and genotype was confirmed by genomic PCR from tail specimens. **(B)** Left: additional examples of live cell images of WT-MeCP2-Halo and G118E-MeCP2-Halo neurons in **Fig. 3A**. MeCP2-Halo protein stained with JFX549 (cyan); DNA stained with SiR-DNA (red). A dashed white line delimits the nuclei. Scale bar: 2 μm. Min and max range values are indicated. Right: neurons dissected from C57Bl/6J wild type mice and infected with a lentiviral vector (LV) to express either Halo-NLS (HaloTag plus a nuclear localization signal) or H2B-Halo, stained with JFX549 (cyan). DNA stained with SiR-DNA (red). Images are a single z-plane acquired from live cells on a laser-scanning super-resolution microscope. A dashed white line delimits the nuclei. Scale bar: 5 μm. Min and max range values are indicated. **(C)** fast-SMT nuclear masking strategy. Neurons are stained with both JFX549 and PA-JF646. The first and last 20 frames are excited with a 561 nm laser to acquire images of the whole nucleus. Max intensity projections of frames 1-20 and 5021-5040 are thresholded to design contours of the nucleus prior (pre) and after (post) the actual SMT movie, acquired with a 633 nm laser excitation and sporadic photoactivation of the PA-JF646 dye with a 405 nm laser (frames 21-5020). The pre- and post-SMT contours are then intersected (∩) to create a final mask (intersect mask) to only retain nuclear signal. Masked SMT movies are fed to the Quot tracking algorithm. Scale bar: 5 μm. **(D)** Distribution of WT-MeCP2-Halo (black line, WT), G118E-MeCP2-Halo (red line, G118E), H2B-Halo (blue line, H2B) and Halo-NLS (green line, NLS) molecules’ jump distances (i.e., the distance travelled by any given molecule in a certain time) between consecutive frames generated by the Spot-On analysis package. *n* indicates the total number of jumps plotted. **(E)** SASPT analysis of fast-SMT data per dissected animal. Heatmap of the marginalized posterior likelihood (probability) of WT-MeCP2-Halo (WT) and G118E-MeCP2-Halo (G118E) diffusion coefficients based on a model of regular Brownian motion with localization error (colors ranging from blue to yellow indicate increasing probability). Each row on the y-axis is a cell; n specifies the total number of neurons analyzed per each dissected pup (#: animal identification number). **(F)** Fraction bound by animal calculated with the SASPT analysis. Value distributions are plotted with a violin plot, where each dot is a single neuron, and the bar indicates the mean fraction bound per animal (animal IDs as in **D**). Black: neurons expressing WT-MeCP2-Halo; red: neurons expressing G118E-MeCP2-Halo; blue: wild type neurons infected with a lentiviral vector to express H2B-Halo; green: wild type neurons infected with a lentiviral vector to express Halo-NLS.

**
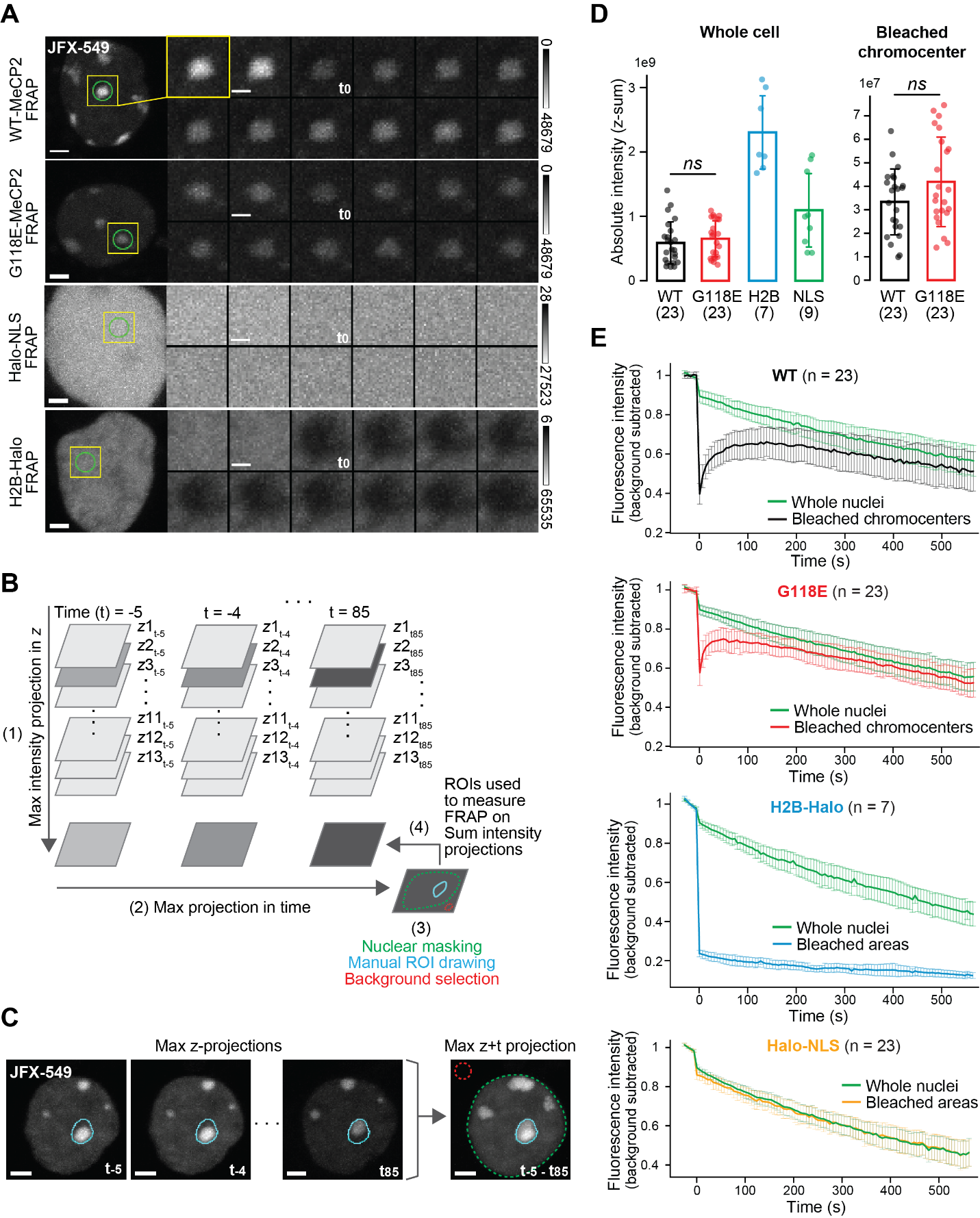
 Supplemental Fig. S7. FRAP measurements of MeCP2-Halo nuclear dynamics in primary cortical neurons. (A)** Representative images of WT-MeCP2-Halo, G118E-MeCP2-Halo, Halo-NLS and H2B-Halo neurons (nuclei) stained with JFX549 for FRAP movies (left, scale bar: 2 μm) and montages of frames 4-15 (right, scale bar: 1 μm). Green circle: bleaching circle (1-μm radius) enclosing the target chromocenter; yellow square, region enlarged in the montage to show bleaching (at t_0_) and recovery dynamics of the target chromocenter. Min and max range values are indicated. **(B)** 3D FRAP analysis schematic. For each cell and time frame (t), we first created a max intensity projection in z (1), followed by a max intensity projection in time (2). This allowed us to manually draw a region of interest (ROI, light blue line) encompassing the target chromocenter as it moved in space and time during the acquisition (3) (see **C** for an actual example). The ROI was then applied to sum z-projections and used for fluorescence recovery measurements (4) as detailed in the Methods section. We used the same max z-t-projections to generate nuclear masks (dashed green line) and select a dark region (dashed red line) for photobleaching correction and background subtraction, respectively. **(C)** Representative example of the 3D FRAP analysis strategy. The chromocenter targeted for bleaching moved significantly form the first (t_-5_) to the last (t_85_) time frame. A projection in time allows us to draw an ROI (cyan shape) that encloses the chromocenter and its movements, identify a dark region for background measurements and draw nuclear masks that consider drift. Scale bar: 2 μm. **(D)** Left: distribution of nuclear JFX549 absolute fluorescence intensity of WT-MeCP2-Halo (WT, black), G118E-MeCP2-Halo (G118E, red) and H2B-Halo (H2B, blue) neurons subject to FRAP. To calculate absolute intensities, for each cell we summed pixel values in z for the first 5 (unbleached) frames and measured their mean pixel fluorescence intensity and nuclear area (in μm). We then multiplied the mean pixel fluorescence intensity by the nuclear area in pixels and plotted the obtained absolute value. Right: MeCP2 (JFX549) absolute fluorescence intensity at chromocenters subject to FRAP in WT-MeCP2-Halo (WT, black) and G118E-MeCP2-Halo (G118E, red) neurons, calculated as in (D) using the chromocenter ROI described in (B) and (C). Each dot is a cell, box and bars are mean and standard deviations, respectively. The number of analyzed cells per sample is specified in parentheses. *ns*: not significant, Mann Whitney U test (two-sided). **(E)** Background subtracted, normalized fluorescence intensity measurements of whole nuclei (green) and bleached regions in WT-MeCP2-Halo (black), G118E-MeCP2-Halo (red), H2B-Halo (blue) and Halo-NLS (yellow) neurons. Absolute fluorescence intensities were calculated from sum z- projections frame by frame. The decaying fluorescence of whole nuclei reflects the acquisition photobleaching. The degree of convergence of the “whole nuclei” and “bleached areas” curves represents the degree of recovery, with full convergence corresponding to complete recovery (e.g., Halo-NLS). n = number of analyzed cells. Curves are mean fluorescence intensities and standard deviations, normalized to the first 5 frames before bleaching.

**
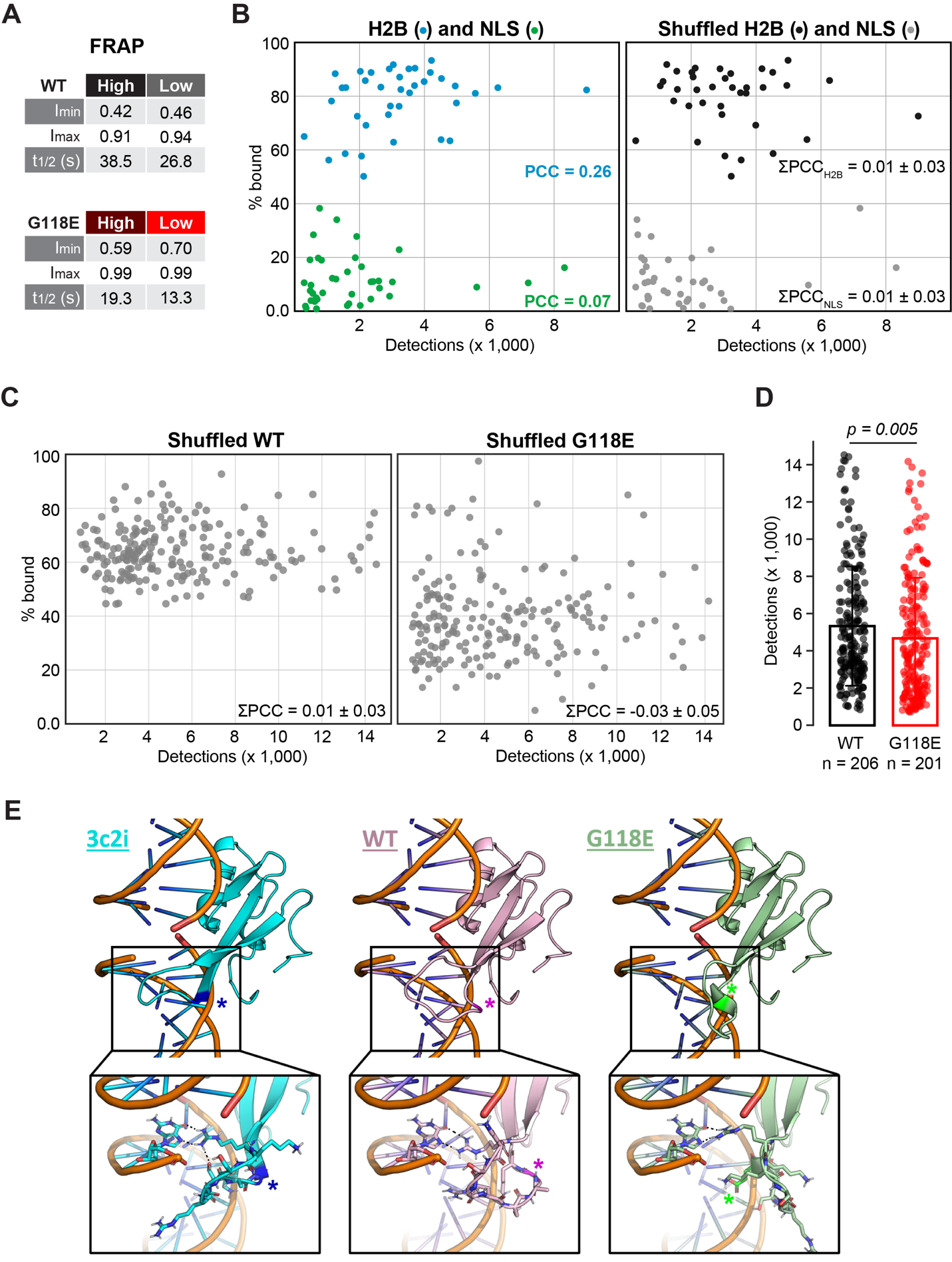
**

**Supplemental Fig. S8. (A)** Tables reporting minimal fluorescence intensity after bleaching (bleach depth, I_min_), maximal fluorescence recovery (I_max_) and time to half recovery (t_1/2,_ in seconds) in WT and G118E neurons expressing high and low MeCP2 levels at the chromocenter subject to FRAP (same as in **Fig. 6A**). **(B)** Left: scatterplot correlating the number of single H2B-Halo (blue) and Halo-NLS (green) molecules detected per cell in fast-SMT experiments (a proxy for protein levels, x-axis) with the fraction of molecules bound to chromatin (% bound, y-axis). Each dot is a single imaged neuron. PCC: Pearson’s Correlation Coefficient. Right: same data but randomized. The indicated Pearson’s Correlation Coefficient (PCC) is the average of 10 iterations +/- the 95% confidence interval. In each iteration, fraction bound values and number of detections were randomly paired, and the overall PCC calculated. **(C)** Representative randomized scatterplots of the number of single WT- or G11E-MeCP2-Halo molecules (stained with the photoactivatable JF-PA646 dye) detected per cell in fast-SMT experiments (a proxy for protein levels, x-axis) versus the fraction of molecules bound to chromatin (y-axis). Each dot is a single imaged neuron (same data as in **Fig. 6B** with randomly paired values). Average PCC +/- 95% confidence interval as in (B). **(D)** Number of single molecules detected in WT (black) and G118E (red) neurons in all fast-SMT movies. Bars are standard deviations from the mean. p: Mann Whitney U test (WT vs. G118E, greater). n = number of imaged cells. **(E)** Rosetta homology modeling starting from a published crystal structure of MeCP2 in complex with methylated DNA (PDB DOI: https://doi.org/10.2210/pdb3C2I/pdb, left, cyan) of the WT (middle, magenta) and G118E mutant (right, green) sequences. Asterisks highlight the amino acid at position 118 (glycine in the 3c21 structure and in the WT protein, and glutamic acid in the G118E mutant protein). Black dotted lines illustrate the preserved hydrogen bonds.

**Supplementary Methods**

*Behavioral assays*

*Open field test*

After habituation in the test room (150 lx, 60 dB white noise), mice were placed in the center of an open arena (40 × 40 × 30 cm), and their behavior was tracked by laser photobeam breaks for 30 min. General locomotor activity was automatically analyzed using AccuScan Fusion software (Omnitech) by counting the number of times mice break the laser beams (activity counts). In addition, rearing activity, the time spent in the center of the arena, entries to the center and distance travelled were analyzed.

*Elevated plus maze*

The elevated plus maze constructed from Plexiglas consists of 2 open arms (35 × 5 cm) and 2 closed arms (35 × 5 × 15 cm) extended from a central platform (5 × 5 cm) that was elevated 50 cm above the floor. After habituation in the test room (700 lx, 60 dB white noise), mice were placed in the center part of the maze facing one of the two open arms. Mouse behavior was video-tracked for 10 min, and the time mice spent in the open arms and the entries to the open arms, as well as the distance travelled in the open arms, were recorded and analyzed using ANY-maze system (Stoelting).

*Three-Chamber test*

The three-chamber apparatus consists of a clear Plexiglas box (24.75 in × 16.75 in × 8.75 in) with removable partitions that separate the box into three chambers. In both left and right chambers a cylindrical wire cup was placed with the open side down. Age and gender-matched C57Bl/6 mice were used as novel partners. Two days before the test, the novel partner mice were habituated to the wire cups (3 inches diameter by 4 inches in height) for 1h per day. After habituation in the test room (700 lx, 60 dB white noise), mice were placed in the central chamber and allowed to explore the 3 chambers for 10 min (habituation phase). Next, a novel partner mouse was placed into a wire cup in either the left or the right chamber. An inanimate object was placed as control in the wire cup of the opposite chamber. The location of the novel mouse was randomized between left and right chambers across subjects to control for side preference. The mouse tested was allowed to explore again for an additional 10 min. The time spent investigating the novel partner (defined by rearing, sniffing or pawing at the wire cup) and the time spent investigating the inanimate object were measured manually.

*PPI*

Mice at 15–16 weeks of life were subjected to acoustic prepulse inhibition. The acoustic prepulse inhibition task consists of presenting the animal with two closely paired sound pulses: a prepulse at +0 dB, +4 dB (74 dB), +8 dB (78 dB), +12 dB (82 dB) and over background followed 100 ms later by a pulse of 120 dB. The amount of startle the pulse induces in the animal is recorded using a startle chamber for mice which records activity for 65 ms after the pulse. The maximum amplitude recorded over the 65 ms is recorded and compared using an ANOVA (genotype) at each prepulse level.

*FootSlip*

Mice were habituated in the test room for 30 min. Each mouse was placed in a footslip chamber consisting of a plexiglass box with a floor of parallel-positioned rods and allowed to move freely for 10 min. Movement was recorded by a suspended digital camera, while footslips were recorded using ANY-maze software (Stoelting Co.). At the completion of the test, mice were removed to their original home cage. Total footslips were normalized to the distance traveled for data analysis.

*Fear conditioning*

A delayed fear conditioning protocol was employed to evaluate hippocampus-dependent contextual fear memory and hippocampus-independent cue fear memory. On day 0 animals were trained in a mouse fear conditioning chamber with a grid floor that can deliver an electric shock (Med Associates, Inc.). This enclosure was located in a sound-attenuating box that contained a digital camera, a loudspeaker and a house light. Each mouse was initially placed in the chamber and left undisturbed for 2 min, after which a tone (30 s, 5 kHz, 80 dB) coincided with a scrambled foot shock (2 s, 0.7 mA). The tone/foot-shock stimuli were repeated after 1 min. The mouse was then returned to its home cage. The context test was assessed in 24 hours. The mice were placed in exactly the same environment and observed for 5 min. The cued fear test was assessed one hour after the context test. The mice were placed in a novel environment for 3 min, followed by a 3 min tone. Mouse behavior was recorded and scored automatically by ANY-maze (Stoelting). Freezing, defined as the absence of all movement except for respiration, was scored only if the animal was immobile for at least 1 s. The percentage of time spent freezing during the tests serves as an index of fear memory. Cued fear memory was the subtraction of freezing time between the tone phase and the no-tone phase.

*Accelerating rotarod test*

After habituation in the test room (700 lx, 60 dB white noise), motor coordination was measured using an accelerating rotarod apparatus (Ugo Basile). Mice were tested for four consecutive days, four trials each, with an interval of 60 min between trials to rest. Each trial lasted for a maximum of 5 min, and the rod accelerated from 4 to 40 r.p.m. The time that it took for each mouse to fall from the rod (latency to fall) was recorded.

*CUT&RUN profiling*

*Nuclear isolation*

Nuclei were isolated from frozen 8-week-old cortex tissues from *Mecp2^G118E/y^*, and wild-type littermates (n=3 for each genotype) using an iodixanol gradient modified from(Mo et al., 2015). Briefly, flash frozen corteces were dropped into a 7 mL dounce homogenizer containing 5 mL buffer HB (0.25 M Sucrose, 25 mM KCl, 2 mM Tricine KOH pH 7.8, 500 μM Spermidine) and dounced 10 x with loose pestle A and 20 x with tight pestle B. Then 320 µL of HB-IGEPAL (HB Buffer +5% IGEPAL CA-630) was added to each homogenizer and dounced 20 x more with tight pestle. Each sample was incubated for 10 min on ice and filtered through a 30 µM filter into a conical tube containing 5 mL of iodixanol working solution (5 volumes Optiprep (Sigma Aldrich, D15556) +1 volume Optiprep Diluent (150 mM KCl, 30 mM MgCl2, 120 mM Tricine-KOH pH 7.8)) and mixed by tube inversion.

To set up the gradient, 4 mL of 40% Iodixanol (3 volumes working solution +1 volumes HB buffer) was added to a 50 mL round bottomed conical tube. Then, 7.5 mL 30% Iodixanol (3 volumes working solution +2 volumes HB) was slowly overlayed on top, followed by 10 mL of the sample containing mixture prepared above. This gradient was spun at 10,000 g for 20 min at 4 °C in a hanging bucket centrifuge (Sorvall Lynx 6000) with ‘decel’ turned off. After centrifugation, nuclei are located at the interface between 30% and 40% iodixanol layers. Iodixanol containing supernatant above the nuclei was slowly discarded with bulb pipette. Approximately 1.5–2 mL of the interface containing nuclei were collected and placed into 2 mL microcentrifuge tube.

The number of nuclei were quantified by taking 20 µL of sample and mixing it with 2 µL 0.2 mg/mL DAPI diluted in HB buffer. After three-minute incubation at RT, the nuclei were diluted 1:10 in buffer HB and counted on a Countess II with DAPI channel to quantify.

*CUT&RUN*

Cleavage Under Targets & Release Using Nuclease (CUT&RUN) was performed on nuclei isolated from above following(Bajikar et al., 2022; Skene & Henikoff, 2017). Briefly, we performed one nuclear isolation per animal and then split the nuclei into two individual tubes for the two antibodies surveyed (MeCP2 and IgG). An individual sample will refer to one antibody from a unique animal.

To activate Concanavalin A coated magnetic beads (Bangs Laboratories Inc, #BP531) for binding, we incubated 25 µL per sample with 3 x volumes binding buffer (20 mM HEPES-NaOH pH 7.5, 10 mM KCl, 1 mM CaCl2, 1 mM MnCl2), rotated at RT for 5 min, and washed 2 x with 1 mL binding buffer. All washes are done by placing microcentrifuge tube on magnetic rack and waiting until solution is clear as beads separated from the solution. Following washes, the beads were resuspended in 50 µL binding buffer per sample.

After bead activation, 200 µL beads were added to nuclei in the iodixanol mixture from each animal and rotated for 10 min at room temperature. Following binding of nuclei to beads, all further processing was done on ice. Bead bound nuclei from each animal was washed 2 x with 1.5 mL wash buffer (20 mM HEPES NaOH pH 7.5, 150 mM NaCl, 500 μM Spermidine [Sigma #S0266], and 0.5% Ultrapure BSA [Invitrogen #AM2618] with 1 tablet of Complete Protease Inhibitor Cocktail [Roche #11873580001] per 50 mL). After the second wash, 250 µL of nuclei bound beads (~400,000 nuclei) in wash buffer were added to individual microcentrifuge tubes corresponding to each antibody.

Supernatant was removed and beads were resuspended in 250 µL of antibody buffer (wash buffer +0.05% Digitonin [Calbiochem #11024-24-1]+2 mM EDTA) containing an individual antibody – rabbit anti-MeCP2 (1:100, Cell Signaling #3456, clone D4F3) and rabbit IgG (1:100, Millipore #12–370). Antibody buffer was added to nuclei bound beads during light vortexing (1100 rpm). Tubes were then placed at 4 °C to rotate overnight. Following rotation, a quick spin on a microcentrifuge was performed to remove liquid from the cap and then washed 2 x with 1 mL Dig-wash buffer (wash buffer +0.05% digitonin). Following the second wash, samples were resuspended in 200 µL dig wash buffer and transferred to PCR strip tubes. Supernatant was removed and beads were resuspended in 100 µL 1 x pAG-MNase (Epicypher #15–1016); 20 x stock pAG-MNase in dig-wash buffer and mixed with gentle flicking. Tubes were placed on nutator for 1 hour at 4 °C. Following incubation, samples were washed with 200 µL dig-wash buffer, transferred to new 1.5 mL microcentrifuge tube, and washed 1 additional time in 1 mL dig-wash buffer.

To initiate cleavage and release of DNA bound fragments, each sample was resuspended in 150 µL of dig-wash buffer while gently vortexing. Samples were placed on ice in 4 °C room for 10 min to equilibrate. To start digestion, while in 4 °C room, 3 µL of 100 µM CaCl2 was added to each tube, quickly flicked, and immediately returned to ice. Following 45-min incubation on ice, 150 µL 2 x STOP 340 mM NaCl, 20 mM EDTA, 4 mM EGTA, 0.05% Digitonin, 100 µg/mL RNAse A (ThermoFisher Scientific #EN0531), 50 µg/mL Glycogen (ThermoFisher Scientific #10814010) and 1 ng E. coli spike-in / sample (Epicypher #18–1401) mixture was added to each sample. Tubes were then incubated at 37 °C for 30 min to digest RNA and release DNA. E. coli spike-in control was used for normalization of CUT&RUN signal due to differences in library amplification and/or sequencing. Supernatant was transferred to new tube and incubated with 1.5 µL 20% SDS and 5 µL 10 mg/mL Proteinase K while lightly shaking at 50 °C for 1 hour. DNA was then purified by phenol-chloroform extraction using Maxtract Tubes (129046, Qiagen) and pellet resuspended in 36.5 µL TE Buffer.

*CUT&RUN next-generation sequencing library preparation*

Library preparation was modified from protocols.IO (dx.doi.org/10.17504/protocols.io.bagaibse) utilizing reagents from the NEB Next II DNA Ultra Kit (New England Biolabs #E7645S) and Unique Combinatorial Dual index kit (New England Biolabs #E6442S,) with modifications outlined below. Input DNA was quantified with Qubit, and 25 µL of CUT&RUN DNA was used for both MeCP2 and IgG samples. Volume of DNA was brought up to 25 μL and 1.5 µL End Prep Enzyme Mixture and 3.5 µL Reaction buffer were added and incubated at 20 °C for 30 min and 50 °C for 60 min. After end prep, 15 µL of NEB Next Ultra Ligation Mastermix, 0.5 µL Ligation Enhancer and 1.25 µL of Adapter (0.6 pmol adapter for MeCP2 and IgG) were added directly to the PCR tube, mixed by pipetting, and incubated for 15 min at 20 °C. Then, 1.5 µL of USER Enzyme is added to each tube. Finally, SPRI select beads (Beckman Coulter # B23318) were used at 1.6 x ratio to remove excess adapter and eluted in 15 µL of TE buffer.

PCR amplification was performed using 13 µL of adaptor ligated fragments, 1 µL of Unique Combinatorial Dual Index (one index per sample), 1 µL of sterile water, and 15 µL 2 x Q5 Master Mix. Fourteen cycles of PCR were performed with 10 s of denaturation at 98 °C and 10 s of annealing/extension at 65 °C. Following PCR amplification, SPRI select beads were used for two-sided size selection; 0.65 x right sided selection was performed first followed by 1.2 x left sided size selection. Sample was eluted in 15 µL TE.

For quality control, each library size distribution was determined by Agilent Tapestation HS DNA 1000 (Agilent Technologies #5067) and concentration was determined by KAPA PCR (Roche #07960140001). Libraries were pooled together at equimolar concentrations and submitted for next-generation sequencing.

*CUT&RUN sequence alignment and visualization*

Our CUT&RUN data analysis pipeline was adapted from CUT&RUN Tools(Zhu et al., 2019). Raw Fastq files were appended together using the Linux cat function. Adapter sequences were removed from sequencing reads using Trimmomatic version 0.36 (2:15:4:4:true LEADING:20 TRAILING:20 SLIDINGWINDOW:4:15 MINLEN:25) from the Truseq3.PE.fa adapter library and kseq(Bolger et al., 2014; Zhu et al., 2019). Confirmation of adapter removal and read quality was performed with fastqc (v0.11.8). Alignment was performed with bowtie2-2.3.4.1 (--dovetail --phred33) to both mm10 (GENCODE GRCm38p6 primary assembly version 18) and the spike-in Ecoli K12 Genomes (GCF_000005845.2_ASM584v2). Bedtools (v2.29.1) was used to convert BAM files to BED files, to remove blacklist (mm9 blacklist lifted over to mm10 and combined with mm10 blacklist downloaded with CUT&RUNTools), and to generate bedgraphs(Quinlan & Hall, 2010). Each sample was normalized to internal Ecoli spike-in utilizing spike_in_calibration.sh as described previously(Meers et al., 2019). Both spike-in normalized bedgraphs for each sample and merged bedgraphs were converted to bigwigs using UCSC bedGraphToBigWig. Integrative Genomics Viewer (IGV) v2.11.1 (Robinson et al., 2011)was used to examine spike-in normalized bigwig tracks at individual loci.

*Cresyl violet (Nissl) staining*

Brains from 16-week-old mice were dissected and freshly embedded and frozen in OCT (VWR). 25µm thick sagittal sections were cut from the frozen tissues and mounted on Superfrost Plus glass slides (Fisher). The slides were dehydrated in 95% and 100% ethanol washes (2-3 each, 2 min each) before an incubation in a 1:1 chloroform:100% ethanol mixture for 20 min to strip lipids. The slides were rehydrated with 95% to 70% ethanol to distilled water (2 min each), then stained with a Cresyl Violet solution 5 min (a filtered solution of: 0.2 g Cresyl Violet; 100 ml ddH2O; 0.3 ml glacial acetic acid; 250 mg sodium acetate). The slides were rinsed in several changes of 95% ethanol to remove excess stain before dehydrating the slides in three changes of 100% ethanol and three changes of xylene (2 mins each). Finally, the slides were mounted using Cytoseal XYL (VWR). The slides were imaged using a Zeiss Axio Scan.Z1 using a 20x0.8 objective.

*Modeling the impact of the G118E Mutation on the structure of MECP2 MBD bound to DNA*

Proteins from PDBs 3C2I, 6C1Y, and 6YWW were confirmed to have identical structures. Alignment of all proteins to each other produced RMSD values > 1.0 Å. PDB 3C2I was arbitrarily selected and the sequence was renumbered. All modeling was performed using PyRosetta (https://doi.org/10.1093/bioinformatics/btq007). Based on visual inspection of the PDB, the loop containing the G118E mutation comprises residues 19-29 in the renumbered PDB (corresponding to residues 110-120). For both the wildtype and mutant protein, the LoopModeler protocol was used to reconstruct the loop from the provided sequence. The MutateResidue function was used to perform the G118E mutation. Residues 19-29 were subjected to an initial build stage, along with centroid and full atom refinement stages, allowing all residues to move and specifying residue 26 as a cut point. Following reconstruction of the loop with the appropriate sequence, the structure was relaxed using the FastRelax protocol using the ‘ref2015’ score function, ‘lbfgs_armigo_nonmonotone’ minimizer, without containing the protocol to the starting coordinates, and again only allowing movement of the loop residues. The resultant wildtype structures showed minimal deviation from the crystal structure (1.32 Å RMSD of loop residues) while the G118E mutant showed a significant deviation in the loop (3.97 Å RMSD of loop residues).

**Supplementary references**

Bajikar, S. S., Anderson, A. G., Zhou, J., Durham, M. A., Trostle, A. J., Wan, Y.-W., Liu, Z., & Zoghbi, H. Y. (2022). MeCP2 regulates Gdf11, a dosage-sensitive gene critical for neurological function. *BioRxiv*, 2022.10.05.510925. https://doi.org/10.1101/2022.10.05.510925

Bolger, A. M., Lohse, M., & Usadel, B. (2014). Trimmomatic: a flexible trimmer for Illumina sequence data. *Bioinformatics*, *30*(15), 2114–2120. https://doi.org/10.1093/BIOINFORMATICS/BTU170

Meers, M. P., Bryson, T. D., Henikoff, J. G., & Henikoff, S. (2019). Improved cut&run chromatin profiling tools. *ELife*, *8*. https://doi.org/10.7554/ELIFE.46314.001

Mo, A., Mukamel, E. A., Davis, F. P., Luo, C., Henry, G. L., Picard, S., Urich, M. A., Nery, J. R., Sejnowski, T. J., Lister, R., Eddy, S. R., Ecker, J. R., & Nathans, J. (2015). Epigenomic Signatures of Neuronal Diversity in the Mammalian Brain. *Neuron*, *86*(6), 1369–1384. https://doi.org/10.1016/j.neuron.2015.05.018

Quinlan, A. R., & Hall, I. M. (2010). BEDTools: a flexible suite of utilities for comparing genomic features. *Bioinformatics*, *26*(6), 841–842. https://doi.org/10.1093/BIOINFORMATICS/BTQ033

Robinson, J. T., Thorvaldsdóttir, H., Winckler, W., Guttman, M., Lander, E. S., Getz, G., & Mesirov, J. P. (2011). Integrative genomics viewer. *Nature Biotechnology 2011 29:1*, *29*(1), 24–26. https://doi.org/10.1038/nbt.1754

Skene, P. J., & Henikoff, S. (2017). An efficient targeted nuclease strategy for high-resolution mapping of DNA binding sites. *ELife*, *6*. https://doi.org/10.7554/ELIFE.21856

Zhu, Q., Liu, N., Orkin, S. H., & Yuan, G. C. (2019). CUT and RUNTools: A flexible pipeline for CUT and RUN processing and footprint analysis. *Genome Biology*, *20*(1), 1–12. https://doi.org/10.1186/S13059-019-1802-4/FIGURES/5
